# The history and geographic distribution of a KCNQ1 atrial fibrillation risk allele

**DOI:** 10.1038/s41467-021-26741-7

**Published:** 2021-11-08

**Authors:** Shannon Hateley, Angelica Lopez-Izquierdo, Chuanchau J. Jou, Scott Cho, Joshua G. Schraiber, Shiya Song, Colin T. Maguire, Natalia Torres, Michael Riedel, Neil E. Bowles, Cammon B. Arrington, Brett J. Kennedy, Susan P. Etheridge, Shuping Lai, Chase Pribble, Lindsay Meyers, Derek Lundahl, Jake Byrnes, Julie M. Granka, Christopher A. Kauffman, Gordon Lemmon, Steven Boyden, W. Scott Watkins, Mary Anne Karren, Stacey Knight, J. Brent Muhlestein, John F. Carlquist, Jeffrey L. Anderson, Kenneth G. Chahine, Khushi U. Shah, Catherine A. Ball, Ivor J. Benjamin, Mark Yandell, Martin Tristani-Firouzi

**Affiliations:** 1 AncestryDNA, San Francisco, CA USA; 2 Nora Eccles Harrison CVRTI, University of Utah School of Medicine, Salt Lake City, UT USA; 3 Division of Pediatric Cardiology, University of Utah School of Medicine, Salt Lake City, UT USA; 4 Cardiovascular Center, Medical College of Wisconsin, Milwaukee, WI USA; 5 Department of Human Genetics, University of Utah, Salt Lake City, UT USA; 6 The Intermountain Medical Center, Murray, UT USA

**Keywords:** Population genetics, Cardiovascular genetics, Genetics research

## Abstract

The genetic architecture of atrial fibrillation (AF) encompasses low impact, common genetic variants and high impact, rare variants. Here, we characterize a high impact AF-susceptibility allele, *KCNQ1* R231H, and describe its transcontinental geographic distribution and history. Induced pluripotent stem cell-derived cardiomyocytes procured from risk allele carriers exhibit abbreviated action potential duration, consistent with a gain-of-function effect. Using identity-by-descent (IBD) networks, we estimate the broad- and fine-scale population ancestry of risk allele carriers and their relatives. Analysis of ancestral migration routes reveals ancestors who inhabited Denmark in the 1700s, migrated to the Northeastern United States in the early 1800s, and traveled across the Midwest to arrive in Utah in the late 1800s. IBD/coalescent-based allele dating analysis reveals a relatively recent origin of the AF risk allele (~5000 years). Thus, our approach broadens the scope of study for disease susceptibility alleles to the context of human migration and ancestral origins.

## Abstract (web summary)

Many rare high-impact variants have been associated with disease, but the origins and functional impact are not always explored. Here, the authors trace the ancestry of a rare high impact atrial fibrillation allele in KCNQ1, and use iPSC-derived cardiomyocytes to characterize the effect of the allele.

## Introduction

Atrial fibrillation (AF) is the most common sustained arrhythmia seen in clinical practice, affecting over 30 million individuals worldwide^1^. AF patients are at significant risk of stroke and twice as likely to die from cardiovascular disease compared to patients in sinus rhythm. The genetic architecture for AF susceptibility has come into focus^2–7^ based on studies of common, low-impact variants derived from genome-wide association studies (GWAS) and rare, high-impact variants discovered in familial forms of AF. Despite the explosion in knowledge of AF genetic architecture, the historical and geographic context of AF-associated risk alleles remains largely unexplored. Towards this end, we adapted a method^8^ for inferring the temporal and geographic patterns of a population using genotypes annotated with genealogical records, to reveal the recent demography and migration patterns of the communities related to a high-impact AF susceptibility allele, *KCNQ1* R231H, segregating in a large Utah family. This same allele has also been shown to cause young-onset AF and Long QT Syndrome (LQTS) in 5 apparently unrelated families^9^ and is annotated as pathogenic in ClinVar^10^, suggesting that this allele may play a role in early-onset AF in North America and Western Europe. Analysis of a genetic relatedness network allowed us to infer carrier status for a set of samples without direct assay of the risk allele, providing a method to detect at-risk individuals. We also compared whole-genome sequence data from distantly-related risk allele carriers to estimate allele age. Thus, we deploy a broad set of analytical tools to characterize a young-onset AF susceptibility allele, *KCNQ1* R231H, from a functional/cellular context, to the broader context of distribution due to human migration, and finally to estimates of when the allele entered the pool of human genetic variation.

## Results

### Clinical ascertainment and genetic evaluation

**Fig. 1:**
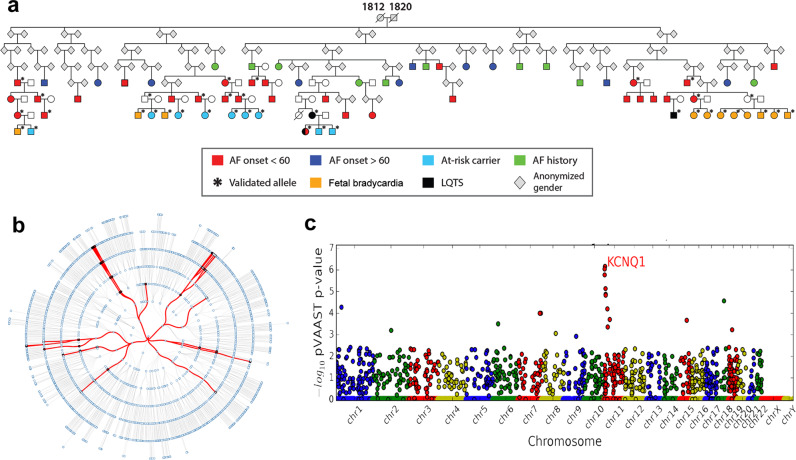
Clinical and genetic evaluation of the AF phenotype. **a** Multi-generational family pedigree with young-onset atrial fibrillation phenotype. Color coded legend describes clinical phenotypes of interest. At-risk carrier refers to younger aged individuals who may yet develop young-onset AF symptoms. **b** Circular pedigree constructed from UPDB data showing 2926 offspring from the founder couple, with successive generations depicted on circular rings. The red lines indicate the arc of disease transmission across generations and denote known carriers of the risk allele, obligate carriers and subjects manifesting young onset AF, LQTS or fetal bradycardia phenotypes. **c** pVAAST identifies *KCNQ1* as the likely AF causative gene. Manhattan plot with chromosome location on *x*-axis and -log10 pVAAST p-value on y-axis, generated by pVAAST analysis of WES data from 5 young-onset AF individuals. Each circle indicates an individual gene. Note the ‘comet tail’ of flanking genes beneath *KCNQ1*; the scores of these genes are elevated because they contain sequence variants linked to the *KCNQ1* R231H allele.

As part of ongoing research efforts to identify high-risk AF pedigrees in Utah, we intersected a Utah Population Database (UPDB)^11–15^ query for AF diagnosis with clinical databases from Primary Children’s Hospital, Intermountain Medical Center and the University of Utah Hospital and Clinic. The result of this query included an 8-generation pedigree encompassing 2926 individuals that was further characterized clinically and genetically (Fig. 1a, b). Based on review of medical records, patients in this pedigree were dichotomized as young-onset AF (age at diagnosis < 60 years; red symbol) or typical AF (age at diagnosis > 60 years; blue symbol). Ten patients with a history of AF lacked documentation of age of onset due to limited or absent medical records (green symbol). Twenty-nine family members in the young-onset AF group presented with a mean age of onset of 32.0 years (range 13–57, Table 1). None of these patients had traditional clinical risk factors for AF (hyperthyroidism, diabetes mellitus, valvular disease, ischemic heart disease, congestive heart failure, cardiomyopathy). Ten family members were documented to have AF onset at > 60 years of age (mean age 74.7 years, range 65–80). Interestingly, none of the offspring of these family members went on to develop young-onset AF (based on UPDB records), suggesting that these family members may have had typical, age-related AF. The presenting electrocardiograms (ECG) of the young-onset AF patients were within normal limits, with 2 exceptions. First, a 13-year-old female presented with paroxysmal AF and a prolonged corrected QT interval (QTc, 563 ms). Her mother was documented to have had an out-of-hospital cardiac arrest while taking a medication known to prolong the QT interval, but subsequent ECGs showed normal QTc intervals. The 13-year-old’s maternal aunt died suddenly and unexpectedly in her sleep in her early 20 s. Second, a 21-year-old distant relative was noted incidentally to have prolonged QTc interval (482 ms) on a routine ECG, but no history of AF. No family member has documentation of a short QT interval. A history of fetal bradycardia was reported in 11 family members.

**Table 1:** Clinical characteristics.

Patient dichotomization by AF age of onset			
	*N*	Age of onset ± SD (years)	Longest QTc ± SD (ms)
AF < 60 years	29	32 ± 13	455 ± 45
AF > 60 years	10	75 ± 7	447 ± 35
**Number of patients with given clinical diagnosis**			
R231H positive	LQTS	Sudden death	Fetal bradycardia
36*	2	1	11

*37 including identical twin who was not genotyped; LQTS, clinical diagnosis of Long QT Syndrome.

The segregation of young-onset AF in the pedigree was consistent with autosomal dominant inheritance, allowing for missing phenotype data and incomplete penetrance. We deployed pVAAST^16,17^, the pedigree-enabled version of the disease-gene finder VAAST^18–21^, on whole-exome sequencing (WES) data from 5 members of the family with young-onset AF and identified the cardiac potassium channel gene *KCNQ1* as the top candidate gene, driven by a damaging genetic variant c.692 G > A (NM_000218.2) encoding a missense substitution R231H (rs199472709, 11-2593251-G > A on GRCh37, frequency 3.19E-5 in gnomAD v2 all genomes, absent from gnomAD v2 exomes and gnomAD v3; Supplementary Methods). The pVAAST Manhattan plot (Fig. 1c) highlights the ‘comet tail’ of flanking genes beneath *KCNQ1* whose scores are likely elevated because they contain sequence variants that co-segregate with the *KCNQ1* R231H allele within the pedigree (Supplementary Table 1). This same allele has also been shown to cause young-onset AF and LQTS in 5 apparently unrelated families^9^. One of the previously reported families^9^, originally ascertained due to profound fetal bradycardia, fits into a branch of our family described here. Cascade family screening identified an additional 18 R231H heterozygous carriers, all of whom were asymptomatic children, some with a history of fetal bradycardia. While not every family member was genotyped, of 39 adult genotype-positive, phenotype-positive or obligate carriers, 31 manifested a young-onset AF or LQTS phenotype, suggesting a 79% penetrance for individuals > 18 years. Eight obligate or known carriers are phenotype negative or pre-onset.

### Functional characterization of the AF risk allele

**Fig. 2:**
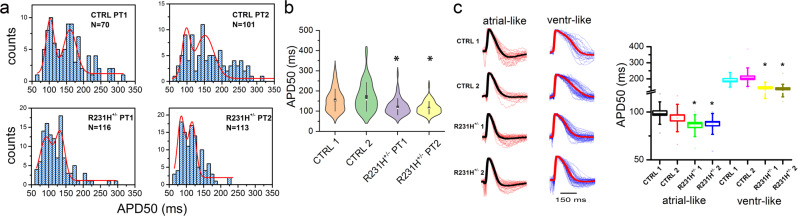
iPSC-CMs derived from heterozygous carriers of *KCNQ1* R231H allele display abbreviated action potential duration. **a** Histograms of APD50 measured from optical action potential recordings from iPSC-CMs derived from healthy controls (CTRL) and heterozygous carriers of *KCNQ1* R231H (R231H^+/−^). Red line depicts data fit to a double Gaussian function. **b** Violin plot of all APD50 values. APD50 values are shorter for R231H^+/−^ iPSC-CMs, as compared to CTRL iPCS-CMs (*p < 0.05 compared to CTRL1 and CTRL2 iPSC-CMs, One-way ANOVA with Bonferroni correction; # of independent cells is displayed in Fig. 2a). Square denotes mean value, circle median and whisker SEM. **c** Atrial- and ventricular-like CMs were defined by optical action potential morphology (triangular vs plateau downslope), guided by the peaks of the Gaussian distributions. **Left panel**, Mean AP trace for atrial- and ventricular-like CMs in black and red, respectively, with individual traces displayed in red and blue. **Right panel**, Box plots comparing APD50 values measured from atrial- and ventricular-like CMs. APD50 values for atrial- and ventricular-like R231H^+/−^ iPSC-CMs were shorter than those of CTRL (*p < 0.05, one-way ANOVA with Bonferroni correction). Atrial-like: CTRL-1 (*N* = 26), CTRL-2 (*N* = 22), R231H^+/−^−1 (*N* = 33), R231H^+/−^−2 (*N* = 32); Ventr-like: CTRL-1 (*N* = 35), CTRL-2 (*N* = 69), R231H^+/−^−1 (*N* = 68), R231H^+/−^−2 (*N* = 70). Small squares show means, horizontal lines show medians, large squares show SEs, error bars show SDs, and hatch marks are ± 99% confidence intervals.

Previous functional characterization of R231H KCNQ1 mutant subunits in a heterologous expression system^9^ revealed a hyperpolarizing shift in the voltage-dependence of current activation, predicted to increase repolarizing current throughout the cardiac action potential, i.e., a gain-of-function effect. To further characterize the functional consequences of this *KCNQ1* AF risk allele, we analyzed the electrophysiological properties of patient-specific cardiomyocytes differentiated from induced pluripotent stem cells (iPSC-CMs) obtained from 2 distantly related (9 meioses separation) heterozygous *KCNQ1* R231H mutation carriers with a young-onset AF phenotype compared to a healthy, non-carrier first-degree relative sibling control and an unrelated control subject. Detailed characterization and validation of iPSC lines and iPSC-CMs are presented in Supplementary Figs. 1 and 2. Optical action potential (AP) recordings of day 30-40 post-differentiation iPSC-CMs were obtained using the near-infrared fluorescent voltage-sensitive dye di-4-ANBDQBS and a high-speed digital camera. We previously demonstrated that di-4-ANBDQBS allows for high-precision optical AP measurements that markedly increase the throughput for electrophysiological characterization of human iPSC-CMs^22^. Optical AP recordings were analyzed for all iPSC-CMs, and for atrial- and ventricular-like cells as classified by their Gaussian distributions and AP morphology (see Methods and Lopez-Izquierdo et al.^22^). Across all groups, heterozygous R231H carriers reveal abbreviated AP duration (APD50, time to 50% repolarization) compared to control values (Fig. 2). Apart from abbreviated APD, R231H heterozygous iPSC-CMs behaved similar to control cells in response to isoproterenol stimulation and step-pacing protocols (Supplementary Figs. 3 and 4).

### AF risk allele distribution and age

To identify additional carriers and more thoroughly assess mutation distribution and history, five AF-risk allele carriers from the large family pedigree shown in Fig. 1a (henceforth referred to as “the original five”) consented to participate in the Ancestry Human Diversity Project and submitted DNA samples to the AncestryDNA genotyping assay. This allowed for comparison of their genotypes to a large number of genotyped samples in the Ancestry database that are consented-to-research, along with per-sample estimates of genetic similarity to broad- and fine-scale populations around the world, and linked family trees.

**Fig. 3:**
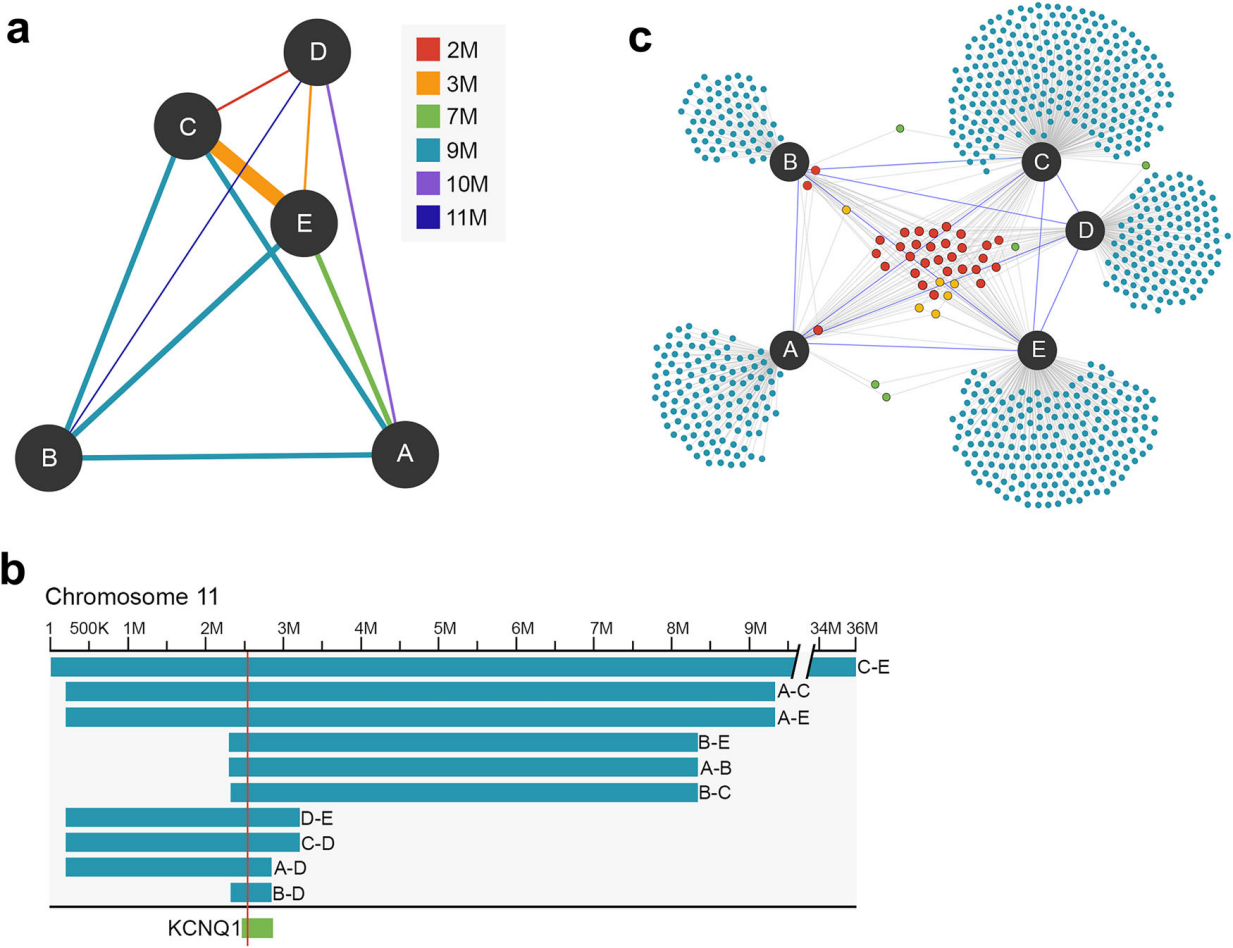
IBD sharing across *KCNQ1* locus. **a** Network showing the amount of IBD shared amongst the original five subjects (denoted by nodes A–E). Distance between nodes corresponds to the number of meioses (M) separating subjects as inferred from each pair’s total amount of shared IBD. Color of edges corresponds to the number of meioses separating subjects according to the known relationships in the pedigree. Edge thickness corresponds to the length of the shared IBD segment across the *KCNQ1* locus. **b** Unphased IBD segments reported across the *KCNQ1* locus amongst the original five subjects (denoted by A–E) are visualized along the genomic map on chromosome 11. Location of the AF risk variant is indicated by the red vertical line. The *KCNQ1* locus spans chr11:2,466,221-2,870,340 hg19. **c** Identification of putative risk allele carriers using IBD-at-locus network. Network nodes represent 824 unphased-IBD-at-locus matches (along with the original five subjects labeled A–E) and edges represent IBD-at-locus sharing. Most samples (782) share IBD-at-locus with only one of the original five (likely due to sharing of the non-mutant haplotype on the homologous copy of the chromosome). Thirty one individuals share IBD-at-locus with all of the original five subjects and are considered putative carriers. Distance between nodes corresponds to the number of separating meiosis events inferred from the proportion of total shared IBD between samples, adjusted to allow for graph readability. Node color represents the d­­­egree of sharing with the original five AF subjects across the *KCNQ1* locus: 1 subject (blue), 2 subjects (green), 3 subjects (orange), and all 5 subjects (red).

Genetic matches to the original five subjects were identified in the AncestryDNA database using an in-house modified implementation^23^ of the identity-by-descent (IBD) detection algorithm, GERMLINE^24^. This method detects “IBD” chromosome segments shared between pairs of individuals, meaning that the chromosome segment has been inherited from a common ancestor, without recombination, by both individuals. As a rule, the extent of genetic relatedness of a pair can be measured by the length and number of the IBD segments shared between them. We labeled as “genetic matches” all samples sharing at least one IBD chromosome segment > 6 centimorgans (cM) anywhere in the genome with at least one of the original five subjects. Out of this set of genetic matches, we labeled “unphased-IBD-at-locus” the samples sharing > 1 cM IBD across the region spanning the *KCNQ1* locus with at least one of the original five subjects. (For convenient reference of terms used, see Supplementary Table 2). We identified 140,722 genetic matches, with 824 of these sharing unphased-IBD-at-locus. The vast majority of the 824 shared unphased-IBD-at-locus with only one of the original five subjects, presumably due to sharing on the non-mutant copy of chromosome 11. The extent of IBD shared amongst the original five confirmed documented relationships between them (Fig. 3a, b, Supplementary Table 3) for relationships between 2 meioses (M2) to 10 meioses (M10); M2 represents the relationship of full-sibling, M10 represents a 4th cousin or similarly distant relation. The most distantly related pair (an M11 according to the pedigree) shared no large (> 6 cM) IBD segments in the genome, and just 1.2 cM IBD across the *KCNQ1* locus. All possible pairs of the original five subjects shared IBD across the *KCNQ1* locus, as expected for samples confirmed to carry the rare risk allele within a single pedigree.

To contextualize these sample sets, we employed in-house developed methods for estimating genetic ancestry at 2 different scales^8,25–27^. For each genotyped sample, we estimated: (1) Broad-scale ancestry—a measure of relatedness from the distant past of hundreds to thousands of years ago defined by AncestryDNA as “genetic ethnicity estimates^25^”. This method detects genetic similarity to a reference panel comprising representatives of continental and subcontinental populations of varying degrees of resolution. (2) Fine-scale ancestry—a measure of more regional, recent relatedness from the past few hundred years defined by AncestryDNA as “Genetic Community assignments^27^”. This method detects genetic similarity to sets of samples sharing recent ancestry that have been annotated with historical and genealogical records. Examples of genetic ethnicities are “Ireland and Scotland,” “China,” and “Ethiopia and Eritrea” and examples of Genetic Communities are “Virginia Eastern Shore Settlers,” “Northern Afro-Jamaicans,” and “Mountain West Mormon Pioneers.” We compared the original five subjects and their genetic matches to the database background estimates, and assessed the significantly (*p* < 0.05) associated genetic ethnicity estimates and significantly (*p* < 0.001) overrepresented Genetic Community assignments.

The original five subjects’ genetic ethnicity estimates (see Supplementary Tables 4–6 for genetic ancestry estimates) were composed of the regions “England, Wales & Northwestern Europe”, “Norway”, “Sweden”, (*p* ≤ 3.8e-2), indicating they share genetic similarity to reference samples with ancestry from these regions. All five subjects were assigned the “Mountain West Mormon Pioneers” (MWMP) Genetic Community, indicating recent shared ancestry to individuals descending from Mormon migrants. No other Genetic Community was assigned to all original five subjects. Similarly, the same genetic ethnicity regions present in the original five subjects were significantly (p < 0.001) associated with the genetic matches set (*n* = 140,722), which also showed a 5.5-fold overrepresentation for the MWMP Genetic Community assignment amongst its members.

**Fig. 4:**
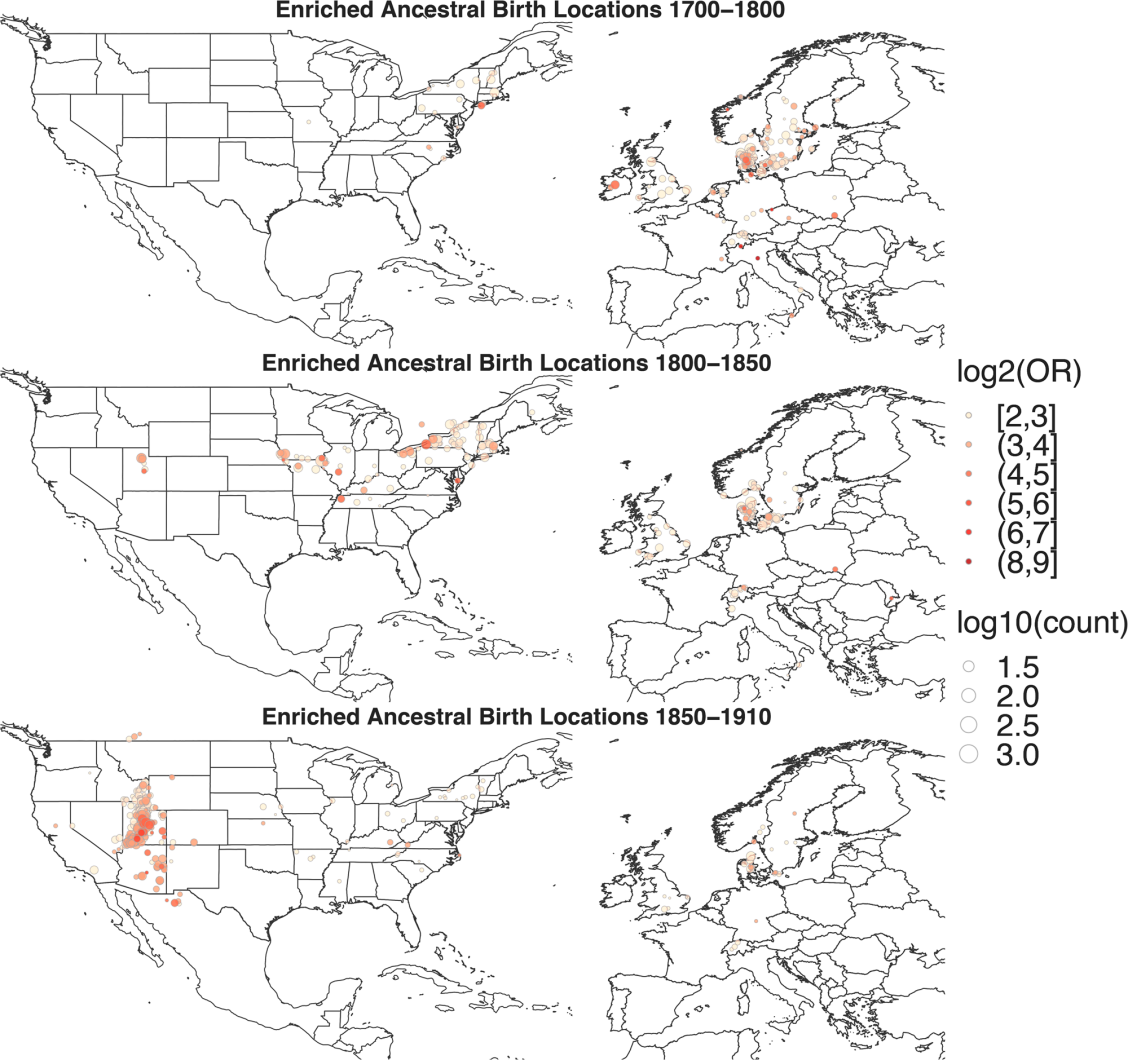
Birth location enrichment maps reveal the historical habitation and migration of ancestors of genetic matches to AF risk allele carriers. Maps of Europe and North America showing enriched birth locations of ancestors of genetic matches to the original five AF subjects. Locations are colored by degree of overrepresentation (odd ratio ≥ 4), with at least 10 unique family trees for each location. Color indicates log2 odds ratio (OR), circle size indicates number of samples per location within a given time period, expressed as log10. The maps reveal the ancestral origin of the genetic matches centered in Denmark, followed by migration to the Eastern United States and then westward across the United States along known Mormon migration routes to Utah.

Next, we used de-identified family trees linked to individuals in the genetic matches set to generate a series of ancestral birth location maps across distinct time periods, allowing for high-resolution ancestry assessment complementary to the broader genetic ancestry estimates. Birth locations and birth years for direct ancestors were extracted from family trees, separated into time periods, and regions associated with ancestral birth records (OR ≥ 4) were plotted (Fig. 4). The resulting maps provide insight into the historical habitations and migrations of ancestors of the genetic matches across generations and confirm reported family history. The maps show a high concentration of enriched ancestral birth records in Denmark at the earliest time period investigated (1700–1800), evidence of migration to the Eastern United States from 1800–1850 with reduced birth records in Denmark and enriched birth record locations scattered across the Eastern United States, and settlement in the Utah region from 1850–1900, recapitulating known Mormon migrations. While our genetic ethnicity reference panel does not include a specific reference set corresponding to Denmark, the genetic ethnicity estimates for Ancestry database samples from individuals with self-reported Danish ancestry are consistent with that of the original five subjects, their genetic matches, and MWMP community sets (Supplementary Methods).

The genetic ancestry estimates and ancestor maps for the genetic matches and MWMP community help to characterize the demographics of the broader population to which the original five subjects belong. To define the history and distribution of the variant itself, and pinpoint with greater resolution any at-risk groups, we sought to distinguish the subset of genetic matches most likely to be AF-risk allele carriers. The unphased-IBD-at-locus genetic matches (*n* = 824) share > 1 cM IBD across the *KCNQ1* locus with at least one of the original five subjects. However, sharing unphased-IBD-at-locus does not necessarily translate to sharing the risk allele, particularly if the IBD sharing is with only one of the original five subjects. Our IBD detection method, which maximizes genome-wide match detection accuracy and computational scalability, evaluates unphased genotypes without discriminating between haplotypes. The subset of genetic matches with unphased-IBD-at-locus, while enriched for the risk allele in comparison to the overall database, likely includes many samples sharing IBD along haplotypes containing the major (non-risk) allele. As we were not able to directly assay the risk variant on our genotyping arrays, we constructed a network of IBD sharing between the original five subjects and their unphased-IBD-at-locus genetic matches (Fig. 3c) to infer “putative carriers” based upon degree of sharing across the *KCNQ1* locus. Using this network, we reasoned that samples sharing unphased-IBD-at-locus with all of the original five subjects are likely carriers of the R231H risk variant. Samples with unphased-IBD-at-locus to just one or a few of the original five likely share IBD along other haplotypes, and hence are unlikely to be R231H carriers given its low population frequency. In this network, 31 samples shared unphased-IBD-at-locus with all of the original five subjects (Supplementary Methods). We considered these 31 samples to be putative carriers. The 782, 5, and 6 samples that shared unphased-IBD-at-locus with 1, 2, or 3 of the original five, respectively, were considered unlikely to harbor the risk allele (Supplementary Methods). We validated this method of inferring carrier status (based upon unphased-IBD-at-locus to multiple known carriers) through Sanger sequencing of a subset of samples across the variant locus. Existing consent status for database samples at the time of Sanger validation allowed for sequencing of 3 putative carriers with unphased-IBD-at-locus to all of the original five subjects. In addition, we sequenced 41 presumed-negative samples that either shared unphased-IBD-at-locus to one of the original five, or belonged to the genetic matches set but without unphased-IBD-at-locus, or were entirely unrelated (Supplementary Table 7). The 3 putative carriers were confirmed by Sanger sequencing to be heterozygous (A/G) at the risk variant locus. All other samples were confirmed to be homozygous for the major allele (G/G) at the locus.

Next, we proceeded to examine the approximate genetic ancestry of the 31 putative carriers from the unphased-IBD-at-locus network. Significant regions (p < 0.05) were “England, Wales & Northwestern Europe”, “Norway”, and “Sweden” (Supplementary Table 5) and 21 out of the 31 putative carriers were assigned to the MWMP community, echoing the ancestry of the larger sets and of the original five subjects. We constructed ancestral birth location enrichment maps for the 31 putative carriers, of whom 16 had available trees with 1618 total ancestral birth data entries. However, 1096 of those entries arise from just 2 of the family trees. Despite significantly lower power due to reduced sample size, the ancestral birth locations of the putative carriers are consistent with those of the genetic matches, suggesting an origin in Northern Europe and subsequent migration across the United States into Utah (Supplementary Fig. 5).

**Fig. 5:**
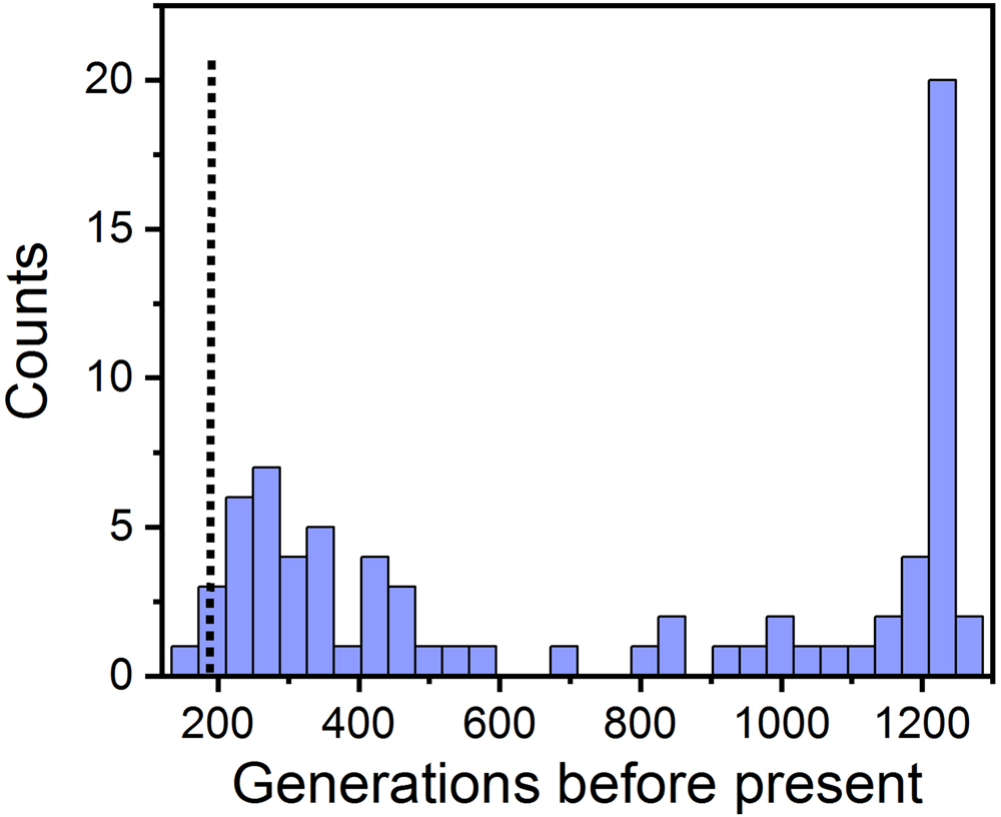
Recent origin of the *KCNQ1* risk allele. Histogram of coalescent estimates of ancestral age of chromosome 11 single nucleotide variants shared by the 2 whole-genome-sequenced *KCNQ1* risk allele carriers, and absent from 99 CEPH-Utah (CEU) 1000 Genomes samples. The dotted line indicates the estimated age of the *KCNQ1* risk allele, corresponding to 182 generations ago (79–719 generations, ±97.5% CI).

Because our exploration of the Ancestry database supported a European origin of the R231H *KCNQ1* allele, we sought to estimate when the allele arose using the Genealogical Estimation of Variant Age (GEVA) method^28^. Briefly, this approach utilizes a hidden Markov model to identify the length of IBD sharing around the allele between pairs of individuals and then estimates the time of the most recent common ancestor. We deployed GEVA on WGS derived from 2 individuals in the AF-risk family pedigree (4 and 5 generations from the founder couple), using 99 CEPH-Utah (CEU) individuals in 1000 Genomes Phase III^29^ as controls. The 2 family members were ascertained as *KCNQ1* R231H carriers prior to pedigree construction, and thus do not violate the assumption that they are random samples with respect to their shared genealogy. We estimate the variant is relatively recently derived, with its origin 182 generations ago (79–719 generations, ± 97.5% CI). To contextualize our results for the AF risk variant, we ran GEVA on every chromosome 11 single nucleotide variant that was shared by the 2 AF carriers and absent from the 1000 Genomes CEU subjects. Out of 39,998 total shared variants, we identified 76 such unique sites and plotted the distribution of their allele ages in Fig. 5. The AF allele is among the youngest such variants on chromosome 11, consistent with being a deleterious mutation that arose recently. Interestingly the *KCNQ1* R231H allele appears as part of a large cluster of doubletons with a similar age, suggesting it was inherited from a very recent common ancestor in the 2 carriers (Supplementary Fig. 6).

## Discussion

Familial forms of AF enabled the identification of rare variants at the high impact end of the AF genetic spectrum. Our study reveals that familial AF can also inform the broader context of distribution due to human migration and estimate when in human history the allele entered the pool of human genetic variation. *KCNQ1* was the first gene implicated in AF susceptibility, with a gain-of-function mutation segregating with the AF phenotype in a large Chinese family^5^. The AF susceptibility allele identified in our Utah family, *KCNQ1* R231H, was previously reported in 5 smaller families of Northern European descent, some of whom also manifested LQTS and fetal bradycardia phenotypes. Like the initial description, the penetrance of the *KCNQ1* risk allele in our family was relatively high, with an estimate of 79% penetrance by adulthood. The R231H mutation localizes to the evolutionarily conserved voltage-sensing domain. Previous functional characterization of the mutant subunits in a heterologous expression system showed a hyperpolarizing shift in the voltage-dependence of channel activation, consistent with a gain-of-function effect. Our analysis of patient-specific iPSC-CMs revealed abbreviated action potential duration compared to controls, corroborating a gain-of-function effect that would be predicted to shorten the effective refractory period, providing a mechanism for AF. However, we observed abbreviated action potentials in both atrial-like and ventricular-like iPSC-CMs, despite no patient manifesting a short QT interval. Features inherent to the human iPSC-CM model system (fetal nature, lack of 3D structure and organization, in vitro differentiation, etc.) may, in part, explain the discrepancy between our in vitro observations and the primary atrial phenotype manifested by patients carrying the AF risk allele.

A limitation of our study is that we did not use isogenic iPSC lines to either introduce the R231H mutation into a well-characterized control iPSC line or correct the mutation in the patient line. Isogenic lines control for the contribution of the genetic background on the phenotype under study, especially in the context of incompletely penetrant disease or small families. However, our large, multigenerational family displays the AF phenotype with an autosomal dominant pattern approaching nearly 80% penetrance. Affected carriers of R231H are separated by 13 meioses in the pedigree, meaning that they share ~ 0.01% of their genomes in common. These facts make it clear that genetic background per se is not a major determinant for the phenotype. This observation is further supported by our experimental data showing that iPSC-CMs derived from distantly related individuals both show abbreviated action potential duration, arguing that this allele causes an aberrant phenotype in the context of diverse genetic backgrounds. Moreover, our observation of abbreviated action potential duration in heterozygous R231H carrier-derived iPSC-CMs is consistent with the gain-of-function effects observed in heterologous expression systems^9^.

Any genetic variant that imparts risk of a potentially lethal, yet treatable, condition provides abundant motivation for the development of methods to identify at-risk individuals. Here, we offer an example of such a method in characterization of the *KCNQ1* R231H mutation and identification of carriers thereof. While portions of our method are unique to the resources of AncestryDNA, much of it can be applied to any large genotype database. Imputing rare variants using methods developed for common variants has proven difficult^30^. The *KCNQ1* R231H mutation, with an estimated frequency of 3.19E-5 (but absent from gnomAD v2 exomes and gnomAD v3), was not amenable to standard imputation and was an ideal candidate for IBD-network-based imputation. This strategy did not require direct assay of the risk mutation in our database because it imputed the mutation for each sample by constructing an unphased-IBD-at-locus network and calculating the degree of relatedness to known carriers. A subset of the putative carriers imputed with this method were validated by Sanger sequencing. Because our strategy does not require phased haplotypes, it is computationally efficient and can be easily deployed on any large genotype database to discover putative carriers for a multitude of risk mutations (see Supplementary Methods). This strategy provides a powerful method for discovery of putative carriers, in the setting of confirmed risk allele carriers.

Broader characterization of the populations harboring a risk variant helps to delineate the variant’s present and historical distribution. Population ancestry estimates, along with ancestral birth location maps, provide a rich account of the group of interest, and help focus public health initiatives on at-risk subpopulations. In our study, these data combine to tell the story of family relatives and the migration of ancestors from Denmark all the way to present day Utah, up to 200 years later. The high odds ratios and number of linked trees supporting the ancestral birth location maps generated support the conclusion that the *KCNQ1* R231H allele traveled to the Intermountain West with the Mormon pioneers. Although underpowered, the same analyses using only the putative R231H carriers point to the same conclusion.

By leveraging whole-genome sequencing data from the carriers we identified, along with publicly available whole-genome sequencing data from 1000 Genomes, we estimated the *KCNQ1* R231H allele to have arisen approximately 200 generations ago, or approximately 5000 years before present, assuming a generation time of 25 years. Compared to other variants on chromosome 11, the *KCNQ1* R231H allele is among the youngest variants shared by 2 *KCNQ1* risk allele carriers and is among the youngest compared more broadly to *KCNQ1* variation worldwide (Supplementary Fig. 7). Since variants subject to negative selection are expected to be younger relative to neutral variants at the same frequency^25^, this is consistent with the deleterious nature of the *KCNQ1* risk allele. Additionally, the amino acid at position 231 is conserved across primates and vertebrates, including zebrafish (Supplementary Fig. 8a). As further *in silico* support of pathogenicity, the risk allele is annotated as “deleterious” / “probably damaging” by the SIFT^31^ and PolyPhen-2^32^ functional impact prediction tools (Supplementary Fig. 8b), and pathogenic by the ensemble prediction tool REVEL^33^ (RankScore 0.994). The mean age of AF onset, 32 years, is within the reproductive time period and thus based on the collective evidence, we hypothesize that the autosomal-dominant, gain-of-function R231H allele is a deleterious mutation likely under negative selection, consistent with recent simulation studies of late onset deleterious alleles^34^.

Here, we present a comprehensive analysis of a young-onset AF risk allele that includes functional characterization, broad- and fine-scale population mapping, and allele age dating. Our approach broadens the scope of study for rare, high impact susceptibility alleles to the context of human migration and ancestral origins to help explain how the Utah population came to be enriched for this clinically important allele. Looking forward, our results also provide a glimpse of how large ancestry databases can be used to better understand the geographic distributions of persons at risk for particular genetic diseases, a necessary prelude to precision health care outreach activities.

## Methods

### Human subjects

Human subject research for the Utah study was conducted under approval from the Institutional Review Boards at the University of Utah and Intermountain Health Care and informed consent was obtained from research participants according to IRB guidelines. All of the AncestryDNA samples included in this study were collected from AncestryDNA customers who agreed to the informed consent for the Independent Review Board-approved Ancestry Human Diversity Project (Quorum Review #26168/1)—an AncestryDNA sponsored research project.

### Utah study patient enrollment and phenotyping

After obtaining informed consent, blood samples were procured from Utah subjects and medical records were reviewed for demographic and clinical data. ECGs were reviewed and QT values were manually measured.

### Exome sequencing data analysis

Raw sequencing data in the form of fastq files (Illumina 101 bp paired-end reads) for each individual were aligned to the human genome reference sequence (GRCh37/hg19, downloaded from http://genome.ucsc.edu) using the Burrows-Wheeler Alignment (BWA) Tool and the MEM algorithm (http://bio-bwa.sourceforge.net/bwa.shtml#13). Variants were called using the best practices protocol for the Genome Analysis Toolkit (GATK) pipeline (http://www.broadinstitute.org/gatk/guide/best-practices). All exomes were joint-genotyped^35^ together with ~190 European individuals (CEU, GBR) from the 1000 Genomes Project. This was followed by Variant Quality Score Recalibration (VQSR), which uses known, high-quality variant sites from HapMap3 and 1000 Genomes to identify and filter potential false positive variant calls. Both variant calling and VQSR were performed separately on INDELs and single nucleotide variants (SNVs). Tranche values were set to 99.5 and 99.0 for SNPs and INDELs, respectively. Whole-genome sequencing (WGS) was performed on two additional patients at a later date. These samples were processed similarly to the exomes and were also joint-genotyped with the ~190 European reference samples described above.

Damaging variants were identified and prioritized using the VAAST2.0 tool developed at the University of Utah^19^. VAAST utilizes an accurate and comprehensive approach to variant prioritization by using the frequency of observing an amino acid substitution in any gene as compared to all variation found in all genes in a large reference population. VAAST can score any coding change and uses phastCon cross-species conservation to further evaluate most variants. VAAST employs a gene-burden test to identify disease-genes using personal genomes data and aggregates prioritization information from each variant in a gene to achieve greater statistical power^19^. Initial VAAST2.0 runs were performed using low-stringency parameters with no specified inheritance pattern and no prevalence cut-off. Subsequent runs were more stringent with enforced dominant inheritance and an allele frequency (prevalence) threshold of 0.05. P-values were determined by permutation, and each run was completed using a two-step process. An initial low permutation run was performed, followed by a high permutation run including only those features that had confidence intervals overlapping significance in the initial run (p ≤ 2.4e–6). Pedigree-VAAST (pVAAST) performs linkage analysis by calculating a novel gene-based LOD score specifically designed for sequence data. The LOD score at each locus is incorporated directly into VAAST to increase the accuracy and decrease the bioinformatic complexity of family-based disease-gene identification efforts^16^. Variants identified by the bioinformatics pipeline were confirmed by sequencing (Sanger) affected and unaffected family members.

### Human iPS cell generation and cardiomyocyte differentiation

While isogenic control iPSCs allow for the direct comparison of the effect of a single mutation in mutant and control cell lines, we elected to use healthy non-mutation carriers as our controls for these experiments. Peripheral blood monocytes (PBMC) were isolated using standard techniques and reprogrammed by infecting cells with lentiviruses expressing OCT4, KLF4, SOX2, and c-MYC from a polycistronic cassette (pHAGE2-TetOminiCMV-hSTEMCCA)^36,37^; further details available in Riedel et al.^38^. Infected blood cells were transferred onto mouse embryonic fibroblast (MEF) feeder cells 3 days post-infection and cultured for additional 7 days in MEF medium supplemented with 10 ng/ml basic human fibroblast growth factor, 5 mg/ml ascorbic acid, and 2 mg/ml Doxycycline. Once small colonies appeared the medium was changed to human embryonic stem cell medium [Dulbecco’s Modified Eagle Media (DMEM)/F12 supplemented with Glutamax, 10 mM NEAA, 25 U/ml penicillin, 25 μg/ml streptomycin, 100 μM β-mercaptoethanol, 20% knockout serum replacement (Invitrogen) with 10 ng/ml bFGF and 2 mg/ml Doxycycline]. Colonies were selected and expanded around day 30 post-infection. After 3 passages on MEF feeder cells, the iPSC clones were transferred to Matrigel (BD Biosciences) coated dishes, cultured and expanded in mTeSR1 according to the manufacturer’s protocol then gradually weaned off of the Doxycycline. Cardiomyocyte differentiation was achieved by the matrix-sandwich method developed by Kamp and colleagues^39,40^. Quality control assays and validation of cell lines are described in (Supplementary Methods).

### Optical action potential recordings

Spontaneously beating human iPS-CMs at day 30–40 post differentiation were plated on coverslips at low density to perform single cell recordings. Human iPSC-CMs were loaded with a near-infrared fluorescent voltage-sensitive dye di-4-ANBDQBS in buffer physiological solution containing (in mmol/l) 126 NaCl, 4.4 KCl, 1.1 CaCl2, 1 MgCl2, 11 glucose, and 24 HEPES (pH 7.4 with NaOH); which was prepared daily from a stock solution of di-4-ANBDQBS dissolved in ethanol. Cells were incubated in 20 µM di-4-ANBDQBS for 5 min and coverslips were then placed on the stage of an inverted microscope, where the fluorescent voltage-sensitive dye was washed out by perfusion with a control buffered solution. Single iPS-CMs loaded with the fluorescent voltage-sensitive dye were excited by a 200 mW red solid-state laser (660 nm), coupled, fluorescent signal was recorded by an electro-multiplied (EM) charge-coupled device (CCD) camera (iXon 860, Andor Technology, Belfast, UK) connected to the video port and equipped with a 700 nm long-pass filter (Omega Optical). All experiments were performed at 36–37° C. For Gaussian distributions and AP morphology, cells were placed at a fixed CL of 800 ms to eliminate any variation in baseline. APD10, APD50, and APD80 were determined as the time at which repolarization of the optical AP achieved 10%, 50%, or 80% of the optical AP amplitude. Optical AP parameters were measured automatically using custom software. For each measurement, the mean value of at least four consecutive APs was calculated. More detailed methods description for optical AP recordings and fluorescent-based optical action potential imaging are available in Lopez-Izquierdo et al.^22^.

### Collection, processing, and genotyping of samples by AncestryDNA

A subset of the *KCNQ1* risk allele carriers from the family pedigree volunteered to participate in the Utah Genome Project / AncestryDNA collaborative research project. AncestryDNA provided a number of test kits to the Utah Genome Project for use. Five participants provided their DNA samples through the AncestryDNA test kits, activated the test kits, and consented to participate in AncestryDNA’s Human Diversity Research Project. DNA samples were processed using standard protocol for AncestryDNA customer-submitted samples (Supplementary Methods). Participating individuals are not indicated in family pedigree for anonymity. Sample collection, processing, genotyping, and quality control of all other AncestryDNA samples used in this study are as described in Supplementary Methods.

### IBD detection

Prior to IBD detection, sample genotypes were phased using Underdog^23^, an in-house modified version of the Beagle^41^ (version 3.3.2) genotype phasing algorithm. Afterwards, related individuals were identified using a proprietary implementation^23^ of the GERMLINE algorithm^24^ (version 1.5.1) to detect long chromosome segments suggestive of inheritance from a recent common ancestor and a proprietary method^23^ to filter out shared DNA that is likely to be identical-by-state (IBS). Our implementation produces the same output as GERMLINE, but offers two computational advantages that allow us to efficiently handle hundreds of thousands of phased genotypes: first, it distributes the computation over a compute cluster; second, it stores the phased genotypes in a database so that new samples can be efficiently compared with previously processed samples. The inputs to our GERMLINE implementation are the phased genotypes, and the genetic distances (in cM) between consecutive pairs of SNPs. The output reproduces that of GERMLINE using the settings listed in Supplementary Table 8. This method uses the phased haplotypes to identify “seed matches” between two samples’ haplotypes, which are then extended until a homozygous mismatch is reached. As such, resulting IBD segment matches are unphased. The minimum threshold for reporting shared DNA was at least one shared segment > 6 cM. For our analysis of unphased-IBD-at-locus segment sharing at smaller segment thresholds (Supplementary Methods), we used our implementation of GERMLINE with smaller thresholds of 1 cM and 0.5 cM and without the IBS filter. IBD network figures were constructed using Gephi version 0.9.2^42^.

### Population genetic ancestry enrichment analyses

The broad-scale population genetic ancestry algorithm uses a hidden Markov model to estimate unphased diploid genetic ancestry across the genome (defined by AncestryDNA as genetic ethnicity estimates) by comparing haplotype structure to a reference panel as described in the Ethnicity Estimate 2019 white paper^25^ and Noto et al.^26^. The reference panel consists of a combination of AncestryDNA customers and publicly available datasets, and is designed to reflect global diversity. Significantly associated genetic ancestries (using “standard” p-value < 0.05, median in-group assigned percentage > 0) were identified by Mann-Whitney U one-sided test using the distribution of genetic ancestry estimates across the AncestryDNA database as baseline compared to the genetic ancestry estimates of the IBD subsets (original five subjects, all genetic matches, and putative carriers).

The fine-scale population genetic ancestry algorithm uses a classification method^43^ that leverages network features to assign individuals to clusters (defined by AncestryDNA as Genetic Communities) in a large network corresponding to recent ancestry and annotated with aggregated historical and genealogical records. Significantly overrepresented Genetic Communities were identified by hypergeometric test using Genetic Community assignments across the AncestryDNA database as baseline compared to the Genetic Community assignments of the IBD subsets (original five subjects, all genetic matches, and putative carriers). P-values were adjusted using Bonferroni correction for the 1073 Genetic Communities tested and results were filtered to adjusted p < 0.001, with > 1.5x overrepresentation in in-group. We selected a stricter p-value cutoff in consideration of the potential bias in overrepresentation for any given community due to the common phenomenon of multiple family members all taking the AncestryDNA test and being assigned the same community. The complete list of Genetic Communities tested can be found at https://support.ancestry.com/s/article/List-of-AncestryDNA-Regions (September 2020).

### Ancestral birth location maps

Publicly-available family trees linked to AncestryDNA tests in this study were used to generate ancestral birth location maps. Detailed usage of genealogical data from publicly-available trees is described in Supplementary Methods of Han et al.^8^. Briefly, Ancestry customers can create family trees and associate their AncestryDNA sample to a node in the tree. We used family trees that the customers had made publicly available, and that were linked to DNA samples. To ensure anonymity, family trees were de-identified, birth location coordinates rounded, birth years aggregated, and records of living people removed. Following all filtering, we obtained a set of 2,902,336 linked family trees. This study focused on the subset of family trees containing direct-line ancestors with birth years between 1700 and 1910. Birth years and locations (map coordinates in latitude and longitude of 2 decimal precision) were extracted from direct-line (parent, grandparent, and so forth) ancestors, aggregated to year ranges (1700-1800, 1800-1850, 1850-1910), and odds ratios calculated per location for each IBD subset (all genetic matches and putative carriers) vs. database background of family tree birth records. For all genetic matches, we plot locations represented in at least 10 unique family trees and having an odds ratio relative to background greater than 4. Because the number of putative carriers is much smaller, we aggregated locations by rounding to the nearest integer latitude and longitude, and required only 2 unique family trees while still requiring the odds ratio relative to background to be greater than 4.

### Sanger sequencing

AncestryDNA samples used in the Sanger sequencing validation study required specific consent to additional processing and hence we only were able to Sanger-validate a subset of the putative carrier set. Research-consented DNA samples from the AncestryDNA storage facility were aliquoted and sent to the Sequencing and Genomics Core Facilities at University of Utah for Sequencing across the *KCNQ1* region spanning the risk variant, using the primer sequences listed in Supplementary Table 9. DNA sample remnants were destroyed by the core facilities after processing in accordance with sample usage protocol.

### Variant age analysis

Two individuals in the pedigree who are AF variant carriers and are distantly related, sharing a common ancestor 4 and 5 generations ago respectively, were whole-genome sequenced. Raw sequencing data for each individual were aligned to the reference human genome (GRCh37/hg19, downloaded from http://genome.ucsc.edu) using the Burrows-Wheeler Alignment (BWA) Tool and the MEM algorithm (http://bio-bwa.sourceforge.net/bwa.shtml#13). The data was statistically phased using eagle v2.4 and HRC reference panel. The *KCNQ1* R231H variant (rs199472709) is not present in the HRC reference panel and was phased manually by visual inspection of IBD sharing. 1000 Genomes Phase III high coverage sequencing data of 99 CEU individuals were combined with the 2 whole-genome sequenced individuals. Since rs199472709 is not present in 1000 Genomes Phase III, we assume the homozygous reference allele for CEU individuals. We then used the ENSEMBL human ancestral alleles to assign ancestral and derived state to every SNP on chromosome 11, keeping only high confidence ancestral alleles (i.e. those where the state matches in multiple non-human apes). To create a control dataset, we found all alleles on chromosome 11 for which the derived allele is present in both carriers who were whole-genome sequenced and absent in the 1000 Genomes CEU individuals. This resulted in 76 additional variants. GEVA (Genealogical Estimation of Variant Age)^28^ was applied on chr11 with fixed recombination rate 1e-08, mutation rate 1.25e-08 and Ne 10,000. We used the posterior mode from the joint clock model, which uses both recombination and mutation to inform allele age estimates, to summarize each site.

### Reporting summary

Further information on research design is available in the Nature Research Reporting Summary linked to this article.

## Supplementary information

Supplementary Information

Reporting Summary

## Data Availability

To protect patient privacy, sequencing data (exome, genome, RNA-Seq) for this study has been deposited into the database of Genotypes and Phenotypes (dbGaP) under accession phs002464.v1.p1 (https://www.ncbi.nlm.nih.gov/projects/gap/cgi-bin/study.cgi?study_id=phs002464.v1.p1) and is available to users upon approval of a Data Access Request (https://dbgap.ncbi.nlm.nih.gov/aa/wga.cgi?page=login). RNA-Seq data for human iPSC lines whose expression patterns were previously corroborated against human embryonic stem cell lines was obtained here: https://www.ncbi.nlm.nih.gov/gds/?term=GSE73211. The human genome reference sequence (GRCh37/hg19) may be downloaded from http://genome.ucsc.edu. The 1000 Genomes Project Phase 3 high coverage sequencing data of 99 CEU individuals used in the variant age analysis can be downloaded from The International Genome Sample Resource website at https://www.internationalgenome.org/data-portal/data-collection/30x-grch38. Ancestry Public Family Trees are available at the Ancestry.com Public Member Trees online database, searchable at: https://www.ancestry.com/search/collections/1030/. While AncestryDNA cannot make the genealogical and genotype data available to the academic community in light of our commitment to customer privacy, we will do our best to accommodate requests regarding methods and summary statistics for the purpose of reproducibility, subject to applicable data use policies. AncestryDNA is interested in pursuing research collaboration opportunities. Please contact Dr. Barry Starr (bstarr@ancestry.com) for methods and summary statistics requests, and for guidelines on submitting a research proposal for consideration.

## Code availability

Software used for the analyses may be obtained from the following sites: GATK: [software.broadinstitute.org/gatk/download/], VEP: [uswest.ensembl.org/info/docs/tools/vep/script/vep_download.html], VAAST, pVAAST. AncestryDNA uses proprietary code that is not publicly available. Detailed descriptions of our methods may be found in the referenced manuscripts and white papers. Suggested settings for running comparable publicly available software are provided in Supplementary Table 8.
