# Supplementary material for: The history and geographic distribution of a KCNQ1 atrial fibrillation risk allele
Source: Nat Commun. 2021 Nov 8;12:6442. doi: 10.1038/s41467-021-26741-7 (PMC8575962; doi:10.1038/s41467-021-26741-7)
Supplement: Supplementary file 1 — Supplementary Information [file 41467_2021_26741_MOESM1_ESM.pdf]

The history and geographic distribution of a KCNQ1 atrial fibrillation risk allele

Hateley, Lopez-Izquierdo, et al.

SUPPLEMENTARY INFORMATION

IPSC Quality Control and Validation 0

RNA-Seq analysis 2

AncestryDNA sample processing 3

Estimating the frequency of the AF susceptibility allele 3

Genetic ethnicity estimates of Danish origin 4

Identifying genetic matches and putative carriers from IBD segments 4

Validating array genotype calls for rare alleles using unphased-IBD-at-locus networks 4

Supplementary Figure 3. Beta-adrenergic response to isoproterenol in R231H$^{+/-}$ iPSC-CMs is similar to control (CTRL) cells. 16

Supplementary Figure 4. Response to increasing pacing rate in R231H$^{+/-}$ iPSC-CMs is similar to control cells. 18

Supplementary Figure 5. Ancestral birth location enrichment maps for putative carriers of AF risk allele. 20

Supplementary Figure 6. *KCNQ1* R231H allele is part of a cluster of recent mutations. 21

Supplementary Figure 7. Estimated age of other *KCNQ1* variants. 22

Supplementary Figure 8. Conservation of *KCNQ1* position 231 amino acid. 23

SUPPLEMENTARY TABLES 24

Supplementary Table 1. List of genes in linkage disequilibrium with *KCNQ1* AF risk allele. 24

Supplementary Table 2. Definitions of selected terms used in manuscript. 25

Supplementary Table 3. IBD amongst original five subjects, with corresponding inferred meiosis events and relationships. 26

Supplementary Table 4. Broad-scale genetic ancestry estimates of the original five subjects.27

Supplementary Table 5. Significant broad-scale genetic ancestry estimates of the Mountain West Mormon Pioneers community, genetic matches, and putative carriers. 28

Supplementary Table 6. AncestryDNA Genetic Communities over-represented in study groups. 29

Supplementary Table 7. Sanger results of selected samples. 31

Supplementary Table 8. Comparable substitute software suggestions. 32

SUPPLEMENTARY REFERENCES 34

SUPPLEMENTARY METHODS

IPSC Quality Control and Validation

Mycoplasma detection

To detect mycoplasma contamination in cell culture, DNA from each cell line was isolated using DNeasy Blood & Tissue Kit (Qiagen, 69504) and a PCR-based Universal Mycoplasma Detection kit (ATCC, 30-1012K) was used according to the manufacturer's instructions. There was no evidence for mycoplasma contamination of any cell lines used in this study.

iPSC authentication using short tandem repeat (STR) profiling

STR analysis provides a rapid and convenient mechanism to authenticate cell lines and detect inadvertent cross-contamination. STR analyses of the cell lines under study were performed using the Geneprint 24 System (Promega, B1870), according to the manufacturer's instructions. The STR profiles of each cell line were screened against the online DSMZ repository (https://www.dsmz.de/fp/cgi-bin/str.html) and found to be unique. The STR profile of each cell line is stored by the Genomics Core Facility at the University of Utah.

Immunofluorescence and flow cytometric detection of human iPSC markers

To validate each iPSC line for pluripotency and detect any unintended differentiation during culture, cells were analyzed for the expression of four hallmark pluripotent stem cell markers: NANOG, Tra-1-60, OCT4 and SOX2. Tra-1-60 is a surface marker, while NANOG, OCT4 and SOX2 are nuclear-localized transcription factors. For both immunofluorescence microscopy and flow cytometry, cells were stained and analyzed in two sets:

Set 1:
Mouse anti-Tra-1-60 IgM (Thermofisher Scientific, MA1-023)
Goat anti-mouse IgM-AF647 (Thermofisher Scientific, A21238)
Mouse IgM isotype control (Thermofisher Scientific, 14-4752-82)

Rabbit anti-Nanog IgG (Thermofisher Scientific, PA1-097)
Goat anti-rabbit IgG-AF488 (Thermofisher Scientific, A11034)
Rabbit IgG isotype control (Thermofisher Scientific, 02-6102)

Set 2:
Mouse anti-OCT4 IgG1 (Thermofisher Scientific, MA1-104)
Goat anti-mouse IgG-AF488 (Thermofisher Scientific, A11001)
Mouse IgG1 isotype control (Thermofisher Scientific, MA1-10405)

Rat anti-SOX2 IgG2a kappa (Thermofisher Scientific, 14-9811-82)
Goat anti-rat IgG-AF555 (Thermofisher Scientific, A21434)
Rat IgG2a kappa isotype control (Thermofisher Scientific, 14-4321-82)

The background signal was determined by the expression level detected using appropriate isotype control antibodies paired to each set.

For immunofluorescence microscopy, cells were fixed with 4% PFA, freshly diluted from 16% PFA (Fisher Scientific, 50-980-487), with 30-minute incubation at room temperature. Cells were washed three times with wash buffer (1% FBS in PBS) by adding 1ml of wash buffer and aspirated after 1 minute. Cells were permeabilized with 0.1% Triton X-100 (Sigma, X100-5ML) for 30 minutes at room temperature and washed three times. Primary antibody incubation was performed in room temperature overnight, followed by three

washes and secondary antibody incubation for 30 minutes. After three washes, nuclei were stained with NucBlue Fixed Cell ReadyProbes Reagent (Thermofisher Scientific, R37606).

For flow cytometry, cells were dissociated with TrypLE Express Enzyme (Thermofisher Scientific, 12605036) and fixed with 4% PFA, freshly diluted from 16% PFA (Fisher Scientific, 50-980-487), with 30 minute incubation at room temperature. Cells were washed with wash buffer (1% FBS in PBS) twice by adding 2ml of wash buffer and centrifuging at 300g for 5 minutes. Cells were permeabilized with 0.1% Triton X-100 (Sigma, X100-5ML) for 30 minutes at room temperature. Cells were washed twice and incubated overnight at room temperature with primary antibodies. After two washes, cells were incubated with secondary antibodies for 30 minutes at room temperature. Cells were analyzed on BD FACSCanto (BD Biosciences) using FlowJo software.

iPSC proliferation assay for human iPSC lines under study
To compare the proliferative rate of IPSCs, each line was stained with carboxyfluorescein succinimidyl ester (CFSE) according to CellTrace CFSE Cell Proliferation Kit manufacturer instructions (Thermofisher Scientific, C34554) and analyzed for CFSE decay over 96 hours of culture. The stained cells were harvested every 24 hours for 4 days and the CFSE level was measured by flow cytometry. NucBlue Fixed Cell ReadyProbes Reagent (Thermofisher Scientific, R37606) was used as the viability stain. The results show similar rates of decay among the four lines, suggesting comparable proliferation rates (Supplementary Figure 1c).

Detection of chromosome aberrations in human iPSC lines under study
To screen our iPSC lines under study for the most common karyotypic abnormalities detected in human iPSC lines, we used the hPSC Genetic Analysis Kit (Stemcell Technologies, 07550), according to the manufacturer's instructions. The qPCR-based kit detects the copy number of the minimal critical regions of commonly mutated genetic loci across chromosomes 1, 4, 8, 10, 12, 17, 18 and X with high specificity and sensitivity through the use of double-quenched probes. All tested loci for the iPSC lines under study were normal with no evidence for deletions or duplications at the commonly mutated loci (Supplementary Figure 1d).

RNA-Seq analysis

RNA was captured using the Illumina TruSeq stranded mRNA library kit with polyA selection. RNA-Seq was performed using Illumina HiSeq 2000 sequencing platform and 125-cycle paired-end reads. Reads were aligned to Hg19 and individual transcript abundance was measured by calculating RPKMs (reads per kb of exon per million mapped reads). RPKM is the sum of the number of reads mapped to all the exons of a transcript, normalized for transcript size and the total sequencing reads for the sample. A multidimensional plot was generated using the leading log_2-fold change (logFC) calculated as the average (root mean square) of the 1000 largest absolute logFCs for genes between the samples. Four iPSC lines were studied: first degree sibling control (CTRL-1), unrelated control (CTRL-2), distant relative R231H heterozygous carrier (R231H-PT2) and a cell line from a young-onset AF patient not related to this family (AF-CTRL). Time points include D0 (stem cell), D3 (cardiac progenitor), D7 (early cardiomyocyte), and D30 (late cardiomyocyte).

Differential gene expression analysis of the RNA-Seq samples was performed using DESeq. A data matrix was created by extracting the normalized counts for the genes annotated to each gene ontology (GO) term: cell cycle (GO:0007049), cell maturation (GO:0048469), cellular senescence (GO:0090398), cardiac muscle cell development (GO:0055013), cardiac muscle contraction (GO:0060048) and fatty acid metabolic process (GO:0006631). Hierarchical cluster analysis of standardized rows was then performed using OriginPro2020b.

Additionally, we compared pluripotent marker gene expression between our cell lines and those of 6 validated human iPSC lines whose expression patterns were previously corroborated against human embryonic stem cell lines (Choi et al, 2015): hiPSC1, hiPSC2, hiPSC3, hiPSC8, hiPSC9 and hiPSC11 (https://www.ncbi.nlm.nih.gov/gds/?term=GSE73211).

AncestryDNA sample processing

All AncestryDNA samples included in this study were collected from AncestryDNA customers. The typical sample collection process for an AncestryDNA customer is as follows: a customer orders an AncestryDNA kit through the AncestryDNA website; the customer collects saliva using kit and returns the saliva sample in stabilizing solution; the customer activates their kit through the website; after kit activation, the DNA sample is processed. Customer genotype data are generated using an Illumina genotyping array with approximately 730,000 SNPs and processed either with Illumina or with Quest/Athena Diagnostics. To ensure quality of each dataset, a sample passes a number of quality control (QC) checks, which includes identifying duplicate samples, removing individuals with a per-sample call rate <98%, and identifying discrepancies between reported sex and genetically inferred sex. Samples that pass all quality-control tests proceed to the analysis pipeline; samples that fail one or more tests must be recollected or manually cleared for analysis by lab technicians. Following sample quality control steps, samples used in this study consented to participate in AncestryDNA's Human Diversity Project.

Estimating the frequency of the AF susceptibility allele

The frequency of *KCNQ1* R231H (rs199472709) is 3.19E-5 in gnomAD v2 genomes[1] due to 1 alt allele in 31,330 total alleles. The alt allele is absent from gnomAD v2 exomes, gnomAD v3, and other variant databases including the Exome Aggregation Consortium[2], the Haplotype Reference Consortium[3], the NHLBI Exome Sequencing Project[4], and the 1000 Genomes Project[5]. The frequency in the Kaviar database[6] is 6.47E-6 due to 1 alt allele in 154,602 total alleles. Frequency estimates for very rare alleles are typically imprecise across independent data sets due to sampling error and the low number of total alt allele observations.

The one individual in gnomAD who harbors *KCNQ1* R231H allele is of Ashkenazi Jewish descent, indicating that either the mutation arose independently or the individual carries the allele through ancestral admixture. Regarding independent mutation, the locus rs199472709, chr11:2593251, is part of a CpG dinucleotide that has been reported as methylated in CD4T cells and monocytes (iMETHYL database[7]). The G>A variant codes as a C>T transition on the opposite strand, which is consistent with 5-methylcytosine deamination that occurs at an order of magnitude higher mutation rate than other nucleotide sites (10^{-7} vs 10^{-8}). Thus, although rare, we cannot rule out independent mutation in the gnomAD individual. However, we consider IBD inheritance through admixture to be the most likely explanation for presence of the allele in the Ashkenazi individual. GnomAD provides global ancestry assignments only, which do not capture the diversity of individuals of Ashkenazi Jewish ancestry, many of whom are admixed with other European ancestries[8]. Our analyses into the estimated age of the allele and the migration mapping of its historical carrier population support a large expansion of the allele's geographic distribution from a single origin. We estimate the mutation to have occurred approximately 200 generations (5000 years) ago, which provides ample time for the variant to spread to other populations through admixture, including the Ashkenazi individual. As no phenotypic information is provided in gnomAD, the AF status of the Ashkenazi individual is unknown.

Genetic ethnicity estimates of Danish origin

According to genealogical history from members of the large pedigree, family ancestors inhabited Denmark prior to immigrating to the United States. While the reference panel used in our genetic ethnicity estimates does not contain representative samples specifically for Denmark, we were able to approximate a Danish reference in the following way: an independent set of genotype samples linked to member-constructed family trees, where all of the oldest direct-ancestor nodes (grandparents or earlier generation) were of Danish origin, were selected, and genetic ethnicity estimates were calculated. The averages of these population-level genetic ethnicity estimates served as a proxy Danish reference. We found the average genetic ethnicity estimates of this proxy to be in line with that of the original five, the genetic matches, and MWMP community sets (Sweden: 36%, England, Wales & Northwestern Europe: 24%, Norway: 23%. Germanic Europe: 16%). This is not to imply that Danes have true ancestry from Sweden, England, Norway, or Germany, but rather that they share genetic similarity to reference samples with ancestry from these regions. Indeed, the process of estimating genetic ancestry based upon genetic similarity is rarely a perfect measure of ancestry because of biases in coverage of the genetic diversity in many populations and gene flow that occurs between populations.

Identifying genetic matches and putative carriers from IBD segments

Genome-wide IBD: Genetic matches for this study were identified using the AncestryDNA match algorithm, which uses a default minimum threshold for reporting detected IBD of at least one shared segment > 6 cM. The 6 cM threshold was chosen in consideration of several factors (e.g. for reporting confident IBD efficiently and at scale), which are described in the AncestryDNA matching whitepaper[9]. The 140,722 matches to the original five subjects, identified in this manner, were labeled “genetic matches”.

Unphased-IBD-at-locus: To identify matches that may share an IBD segment spanning the variant locus < 6 cM due to historical recombination events near the variant, we ran a second iteration of IBD detection, with a shorter shared segment length threshold of 1 cM, on the set of 140,722 genetic matches along chromosome 11 to detect unphased IBD across the *KCNQ1* locus with greater resolution. From this second iteration of genetic matches, 824 samples were labeled “unphased-IBD-at-locus” because they shared > 1 cM IBD with at least one of the original five along the region spanning the *KCNQ1* locus. When matching the original five subjects to each other, the lower 1 cM threshold was required to detect a shared at-locus segment between the most distantly related pair of the five (M11 according to the pedigree). As confirmation of detected IBD, we manually phased, by visual inspection of genotypes, the original five samples and the samples used in Sanger validation. There is a known phenomenon that the accuracy of IBD detection drops with decreasing IBD segment length[10]. Additionally, as match-seed segments are extended in an unphased manner until a homozygous mismatch is reached, reported IBD segments may extend somewhat past regions of actual IBD - that is to say, some noise is expected at reported segment ends. To help control for such factors occurring with short IBD segments, we required further characterization of all unphased-IBD-at-locus samples using an IBD network.

Putative carriers: We constructed an IBD network (Main text, Fig. 3c) of the unphased-IBD-at-locus set (n=824) to differentiate between putative carriers and samples sharing IBD on non-mutant haplotypes. The unphased-IBD-at-locus genetic matches underwent one additional IBD detection analysis using a threshold of 0.5 cM, to identify samples sharing a short IBD segment with any remaining one of the original five subjects. This method identified 31 subjects matching all 5 Utah samples who are considered to be putative carriers of the risk allele.

Validating array genotype calls for rare alleles using unphased-IBD-at-locus networks

Unphased-IBD-at-locus networks can additionally be used to validate genotyping array marker performance. Rare alleles are more likely to be called incorrectly on genotyping arrays due lack of training samples for the

array cluster plot models[11]. Analyses of rare mutations, especially mutations with population frequencies that fall within the range of genotyping error, such as *KCNQ1* R231H, may benefit from a computational validation step using IBD networks as described in this study, i.e. if an array marker is correctly calling a rare allele, then samples with that allele should (if the mutation is from a single founder population) share IBD-at-locus with one another.

SUPPLEMENTARY FIGURES

Supplementary Figure 1

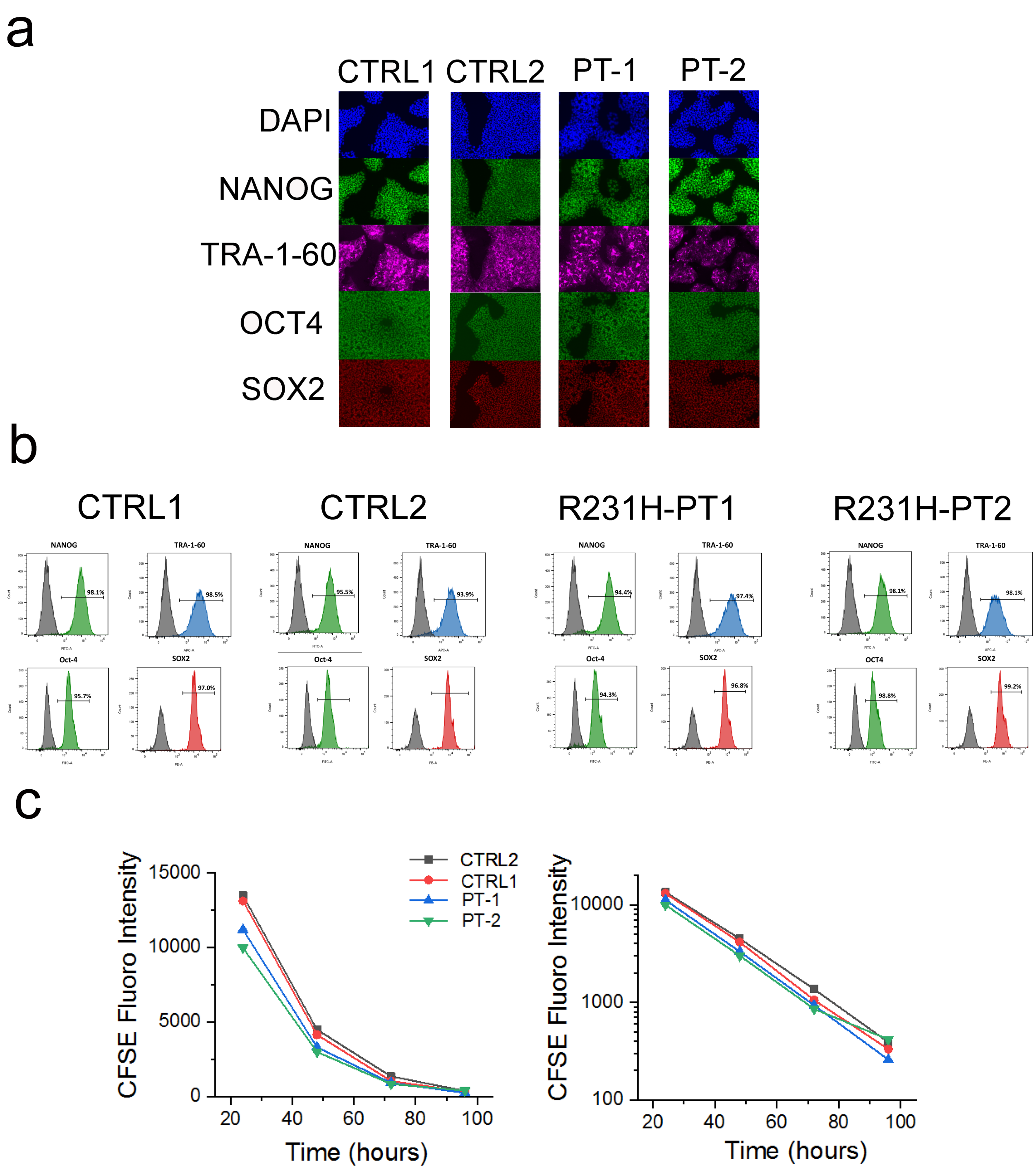

d

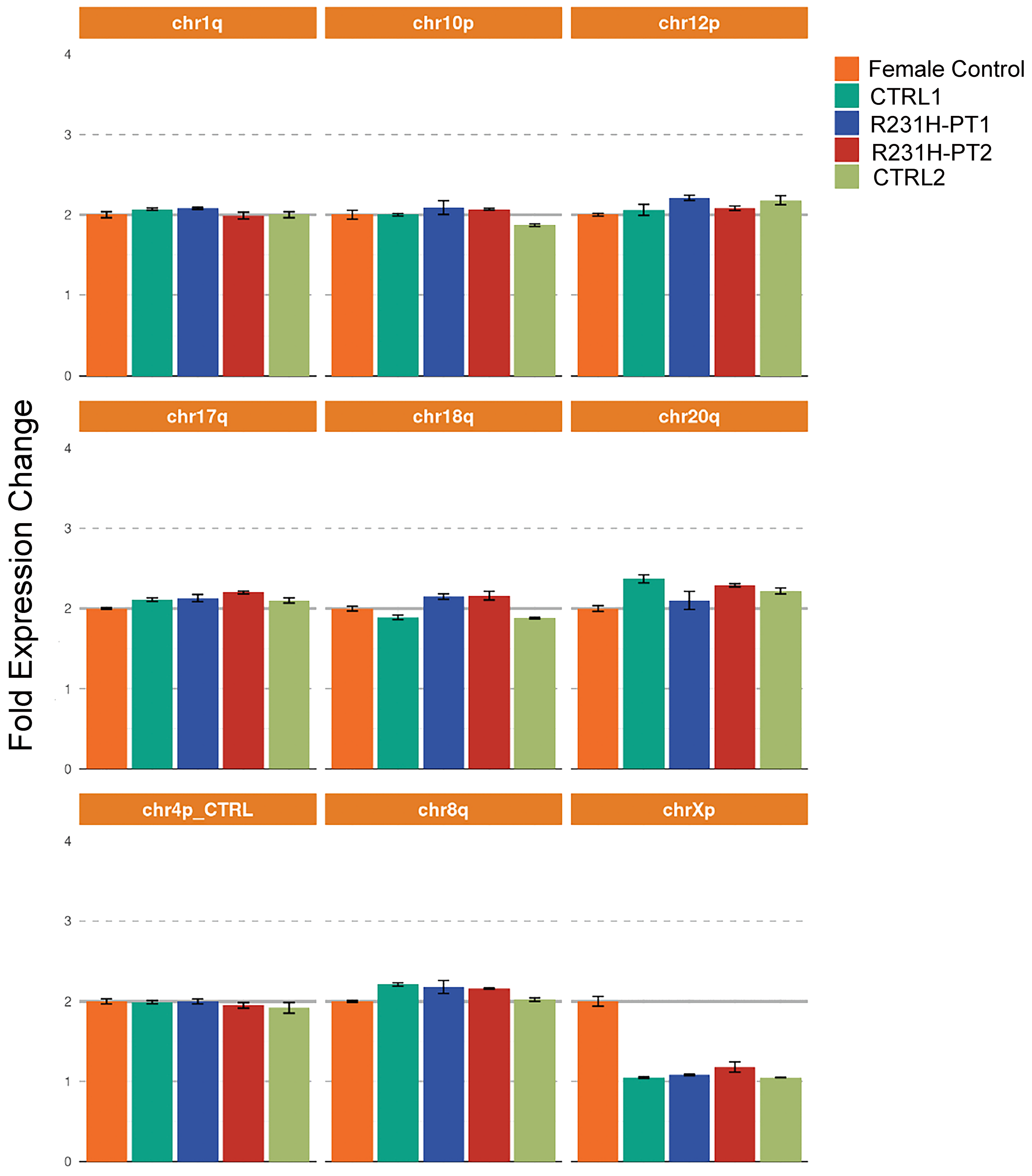

chr1q
chr10p
chr12p
chr17q
chr18q
chr20q
chr4p_CTRL
chr8q
chrXp
Fold Expression Change
0
1
2
3
4
Female Control
CTRL1
R231H-PT1
R231H-PT2
CTRL2

Supplementary Figure 1. Characterization of human iPS cell lines under study. a, Immunohistochemistry staining for DAPI (to stain nuclei) and key markers of pluripotency NANOG, TRA-1-60, OCT4 and SOX2, showing expected localization of markers to nuclei (NANOG, OCT4, SOX2) or cytoplasm (TRA-1-60). 20X magnification. **b**, Flow cytometry data for each iPS cell line under study, showing the percentage of cells expressing the key markers of pluripotency. **c**, iPS cell line proliferation as assayed by the decay of CFSE demonstrating similar rates of proliferation across the cell lines under study. Both panels are identical except for the log10 scale of right panel showing linear decay of log10-fluorescent signal over the study period. Each line represents the data from a single experiment. CFSE, carboxyfluorescein succinimidyl ester; Fluor, fluorescence. **d,** To rule out the presence of the most commonly reported human iPS cell line karyotypic anomalies, we used a qPCR based hPSC Genetic Analysis Kit (Stemcell Technologies). None of the lines under study carried any of the common chromosome anomalies across chromosome regions 1, 4, 8, 10, 12, 17, 18, 20, X, as noted by the diploid values. Regarding the X chromosome, the cell lines in this study are derived from male subjects and thus they carry one copy of the X chromosome; the manufacturer's control sample is female. Experiments were performed in triplicate. The error bars denote standard deviation.

Supplementary Figure 2

a

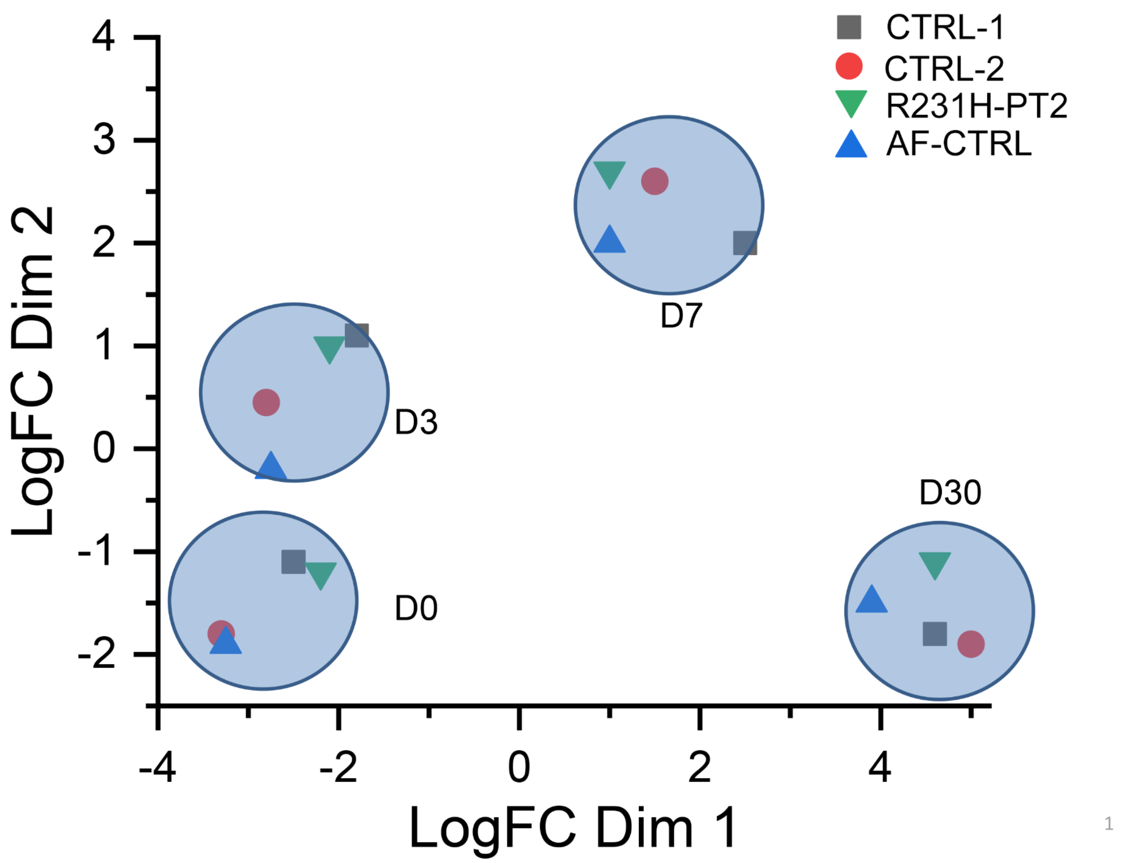

b

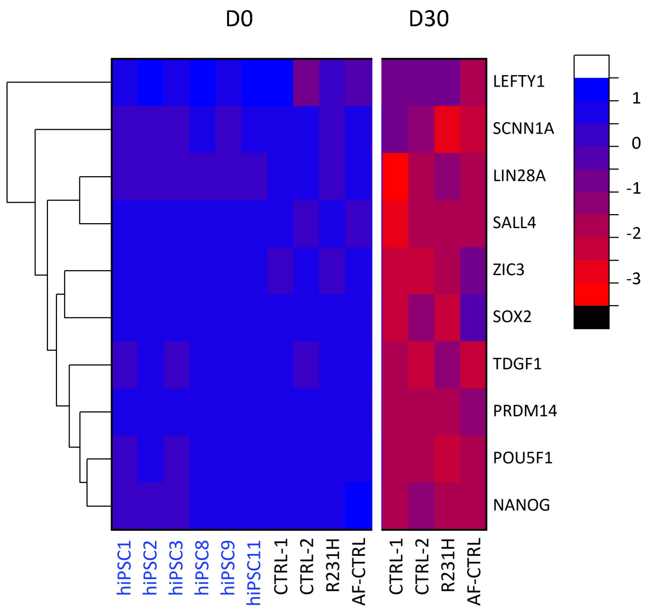
D0
D30
LEFTY1
SCNN1A
LIN28A
SALL4
ZIC3
SOX2
TDGF1
PRDM14
POU5F1
NANOG
1
0
-1
-2
-3
hiPSC1
hiPSC2
hiPSC3
hiPSC8
hiPSC9
hiPSC11
CTRL-1
CTRL-2
R231H
AF-CTRL
CTRL-1
CTRL-2
R231H
AF-CTRL

C

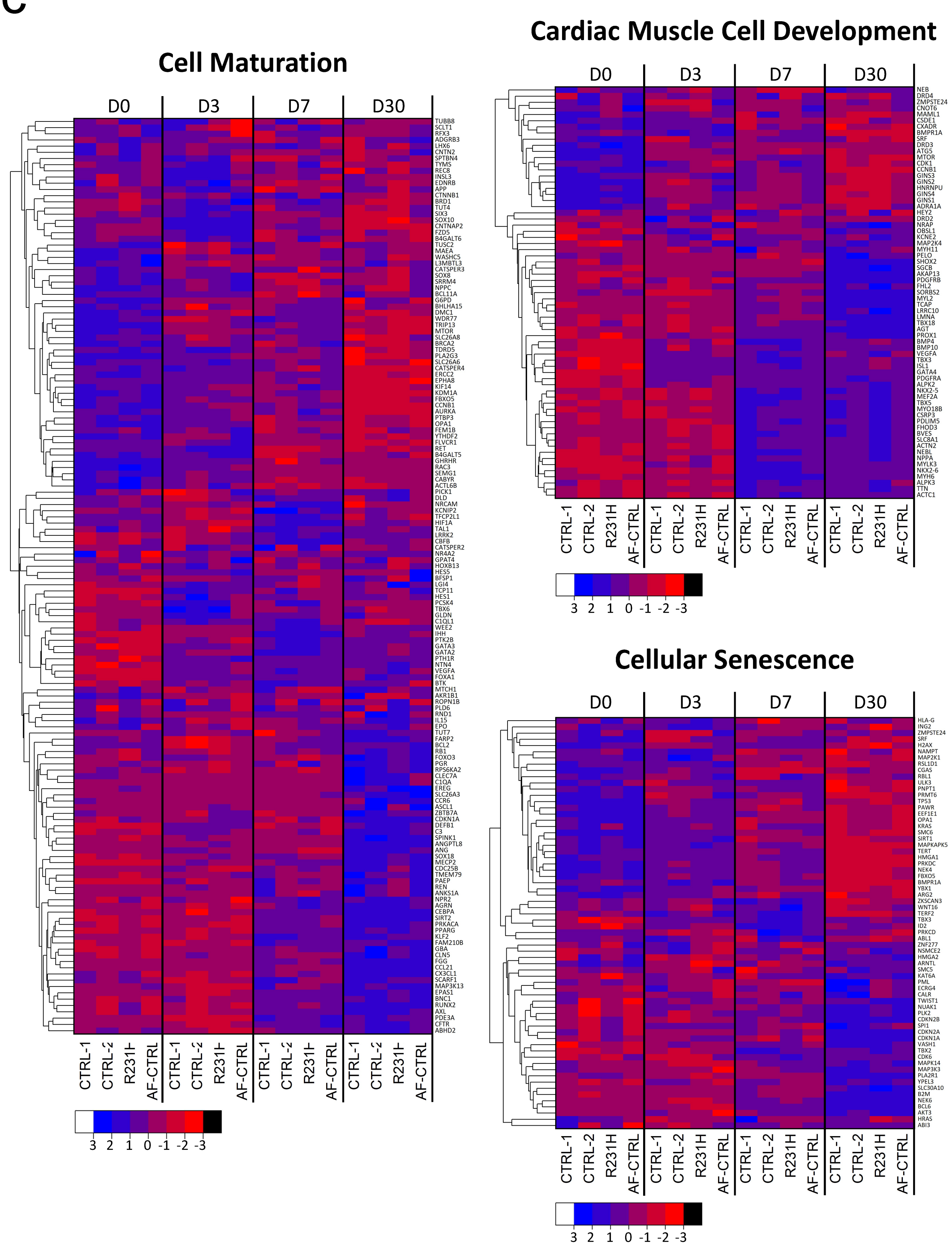
Cell Maturation
D0
D3
D7
D30
CTRL-1
CTRL-2
R231H
AF-CTRL
3 2 1 0 -1 -2 -3
Cardiac Muscle Cell Development
D0
D3
D7
D30
CTRL-1
CTRL-2
R231H
AF-CTRL
3 2 1 0 -1 -2 -3
Cellular Senescence
D0
D3
D7
D30
CTRL-1
CTRL-2
R231H
AF-CTRL
3 2 1 0 -1 -2 -3

d

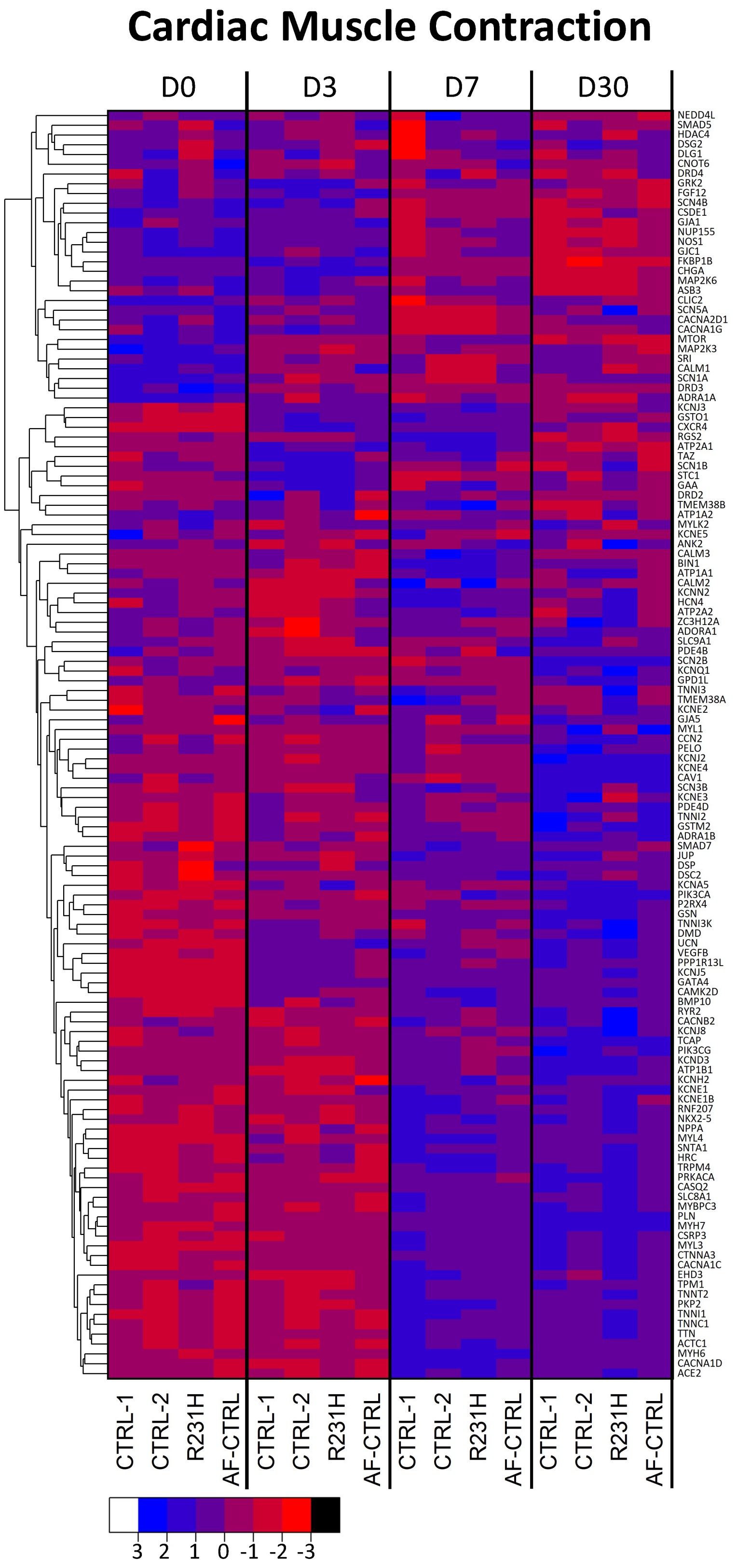
Cardiac Muscle Contraction
D0
D3
D7
D30
NEDD4L
SMAD5
HDAC4
DSG2
DLG1
CNOT6
DRD4
GRK2
FGF12
SCN4B
CSDE1
GJA1
NUP155
NOS1
GJC1
FKBP1B
CHGA
MAP2K6
ASB3
CLIC2
SCN5A
CACNA2D1
CACNA1G
MTOR
MAP2K3
SRI
CALM1
SCN1A
DRD3
ADRA1A
KCNJ3
GSTO1
CXCR4
RGS2
ATP2A1
TAZ
SCN1B
STC1
GAA
DRD2
TMEM38B
ATP1A2
MYLK2
KCNE5
ANK2
CALM3
BIN1
ATP1A1
CALM2
KCNN2
HCN4
ATP2A2
ZC3H12A
ADORA1
SLC9A1
PDE4B
SCN2B
KCNQ1
GPD1L
TNNI3
TMEM38A
KCNE2
GJA5
MYL1
CCN2
PELO
KCNJ2
KCNE4
CAV1
SCN3B
KCNE3
PDE4D
TNNI2
GSTM2
ADRA1B
SMAD7
JUP
DSP
DSC2
KCNA5
PIK3CA
P2RX4
GSN
TNNI3K
DMD
UCN
VEGFB
PPP1R13L
KCNJ5
GATA4
CAMK2D
BMP10
RYR2
CACNB2
KCNJ8
TCAP
PIK3CG
KCND3
ATP1B1
KCNH2
KCNE1
KCNE1B
RNF207
NKX2-5
NPPA
MYL4
SNTA1
HRC
TRPM4
PRKACA
CASQ2
SLC8A1
MYBPC3
PLN
MYH7
CSRP3
MYL3
CTNNA3
CACNA1C
EHD3
TPM1
TNNT2
PKP2
TNNI1
TNNC1
TTN
ACTC1
MYH6
CACNA1D
ACE2
CTRL-1
CTRL-2
R231H
AF-CTRL
CTRL-1
CTRL-2
R231H
AF-CTRL
CTRL-1
CTRL-2
R231H
AF-CTRL
CTRL-1
CTRL-2
R231H
AF-CTRL
3 2 1 0 -1 -2 -3

e

Fatty Acid Metabolic Process

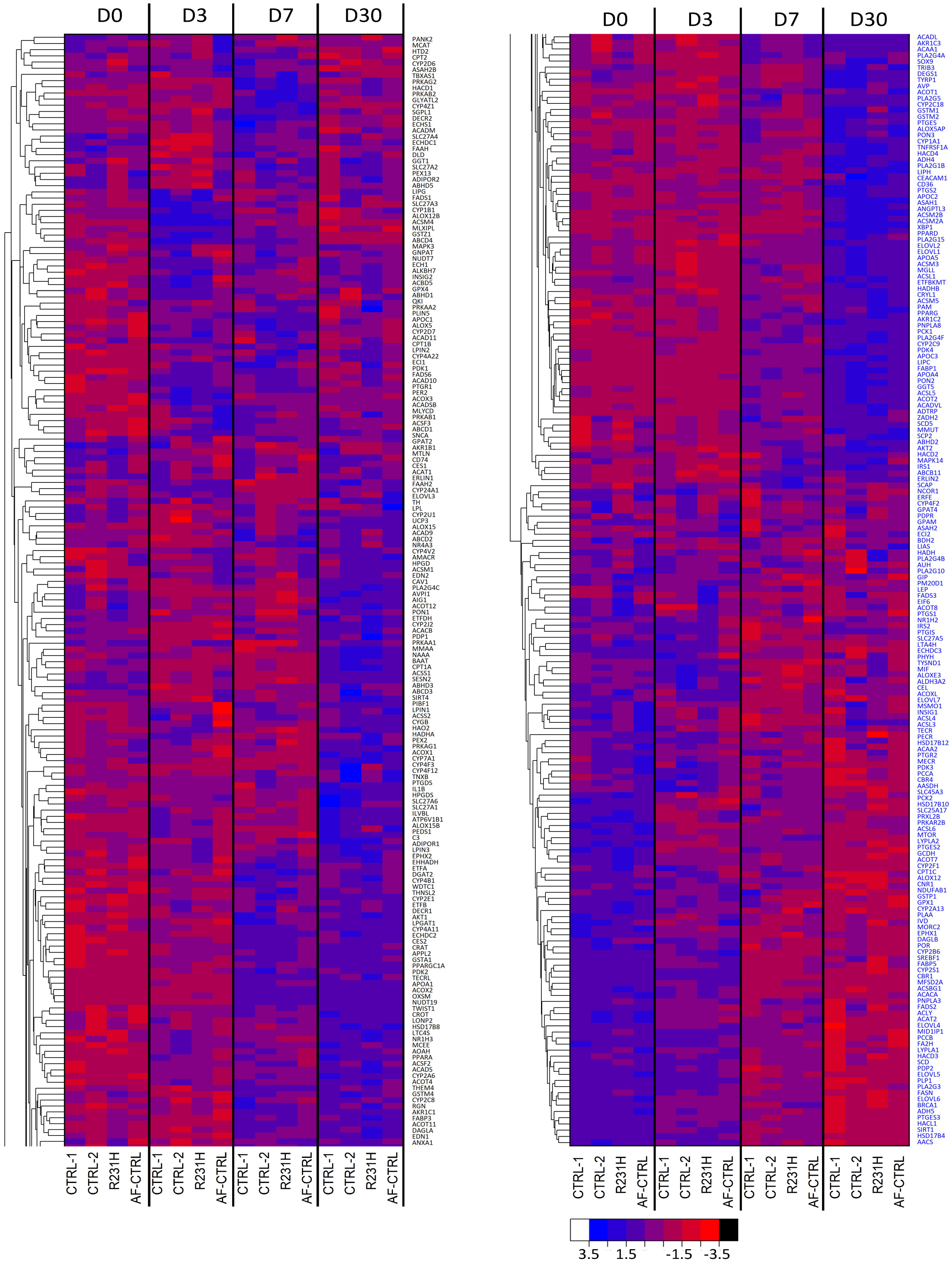

f

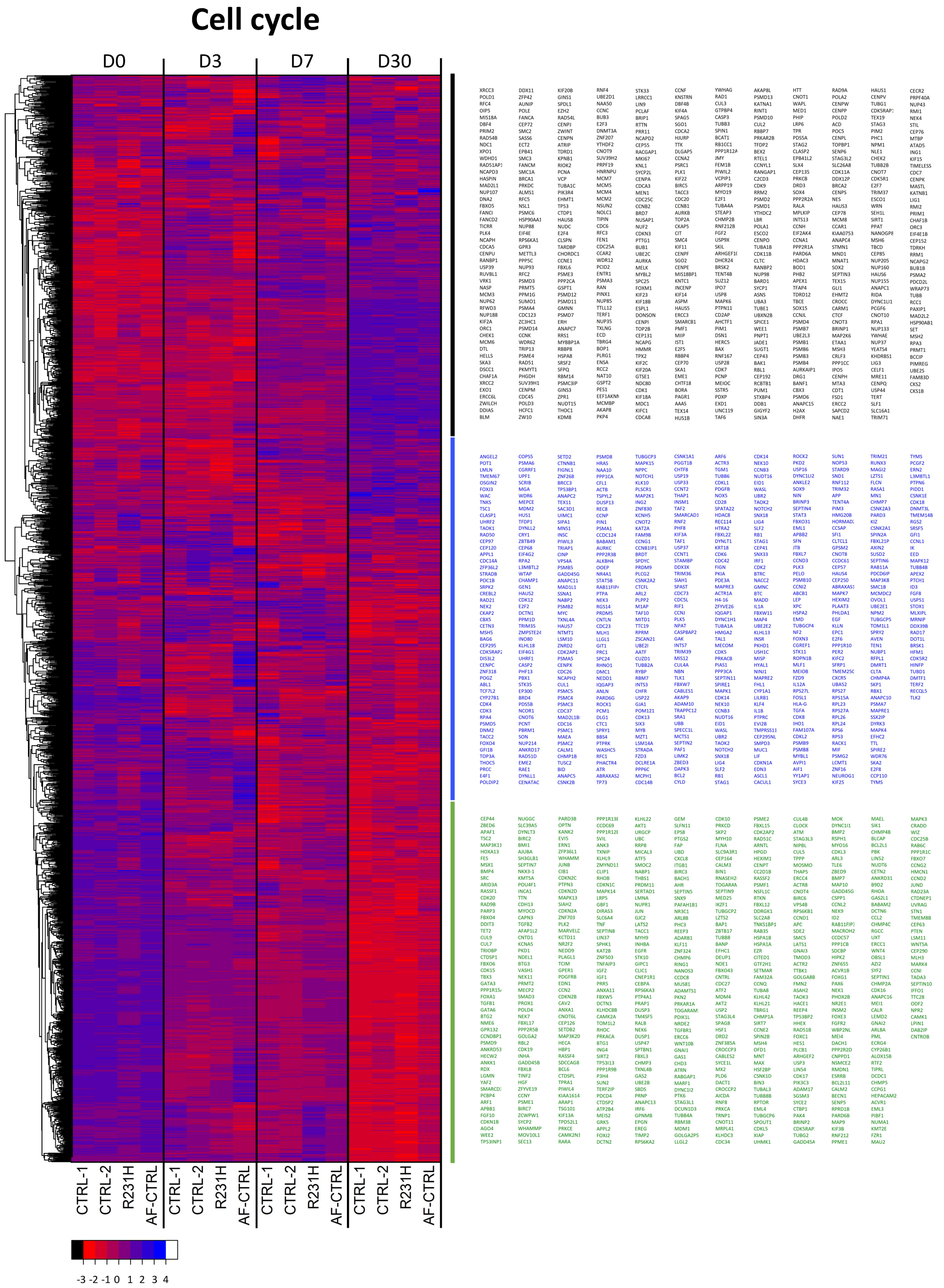
Cell cycle
D0
D3
D7
D30
CTRL-1
CTRL-2
R231H
AF-CTRL
CTRL-1
CTRL-2
R231H
AF-CTRL
CTRL-1
CTRL-2
R231H
AF-CTRL
CTRL-1
CTRL-2
R231H
AF-CTRL
-3 -2 -1 0 1 2 3 4

g

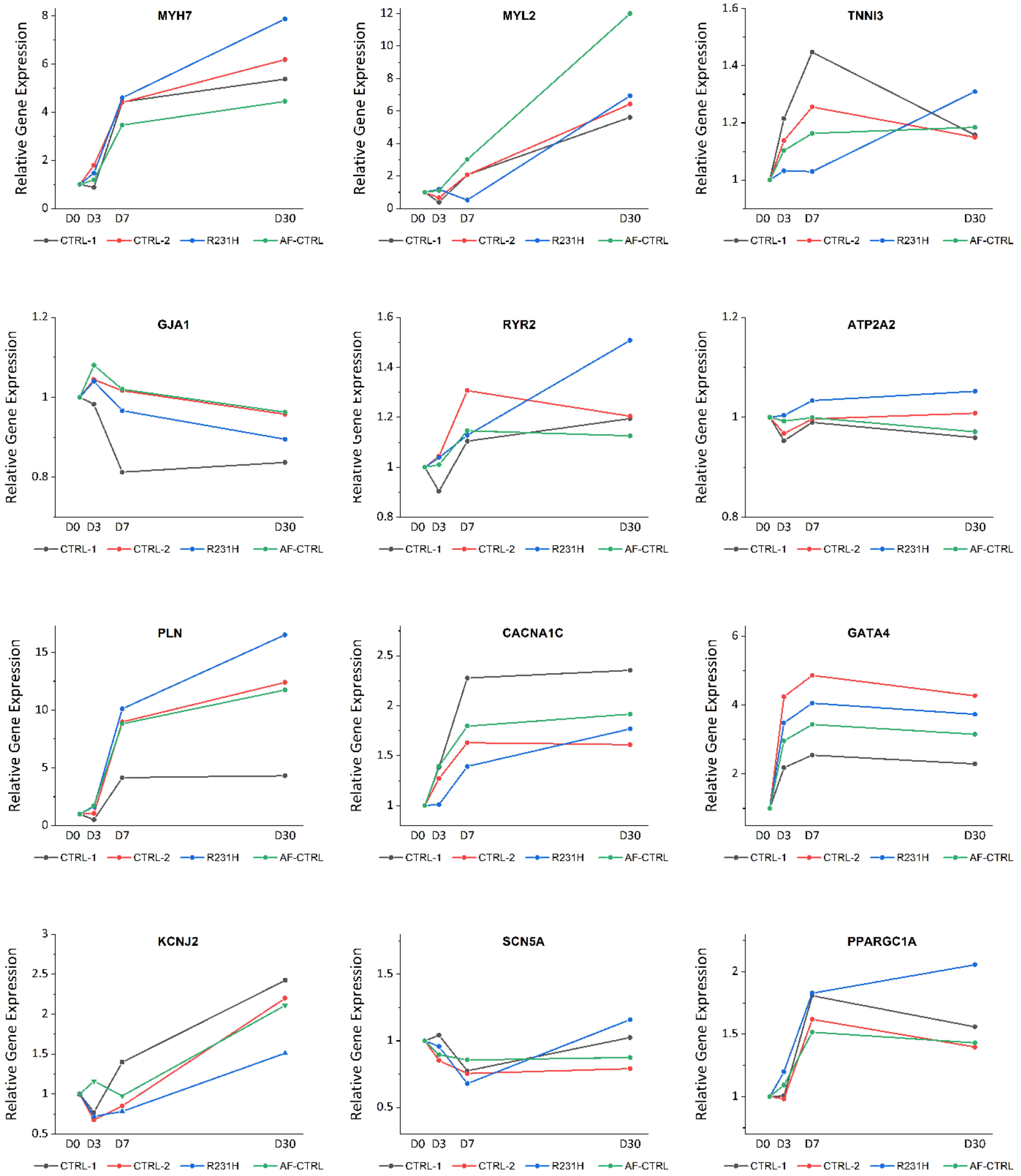
MYH7
MYL2
TNNI3
GJA1
RYR2
ATP2A2
PLN
CACNA1C
GATA4
KCNJ2
SCN5A
PPARGC1A
Relative Gene Expression
D0
D3
D7
D30
CTRL-1
CTRL-2
R231H
AF-CTRL

Supplementary Figure 2. Time course of iPSC-CM gene expression assayed by RNA-Seq. a, Time course of global gene expression during differentiation of iPSC-CMs. The first 2 principal components are plotted from the leading log_2-fold change (LogFC) analysis of the top 1000 variable genes assayed using RNA-Seq from 4 iPSC lines: first degree sibling control (CTRL-1), unrelated control (CTRL-2), distant relative R231H heterozygous carrier (R231H-PT2) and a cell line from a young-onset AF patient not related to this family (AF-CTRL). Time points include D0 (stem cell), D3 (cardiac progenitor), D7 (early cardiomyocyte), and D30 (late cardiomyocyte). Each data point represents a single RNA-Seq experiment. **b**, Comparison of key pluripotent stem cell genes between the iPSC lines under study and human iPSC lines validated against human embryonic stem cell lines (Choi et al, 2015). The heat map demonstrates that relevant pluripotent gene expression in our lines is generally similar to that of published iPSC lines validated against the gold-standard embryonic stem cell lines. **c-f**, Heatmaps showing expression of genes related to cell maturation (GO:0048469), cardiac muscle cell development (GO:0055013), and cellular senescence (GO:0090398) (**c**), cardiac muscle contraction (GO:0060048) (**d**), fatty acid metabolic process (GO:0006631) (**e**) and cell cycle (GO:0007049) (**f**), across time points D0 (stem cell), D3 (cardiac progenitor), D7 (early cardiomyocyte), and D30 (late cardiomyocyte) for the 4 cell lines displayed in Supplementary Figure 2a. The heat maps indicate that the four cell lines globally express similar genes across the time points assayed, consistent with their differentiation along the cardiomyocyte pathway. Each line represents a single gene in the GO category. The color intensity represents the fold increase (blue) or decrease (red) expression, as listed in the legend bar. **g**, Time course of relative gene expression for selected cardiac genes across the time points of days 0, 3, 7 and 30 post-differentiation. Each data point represents data extracted from a single RNA-Seq experiment.

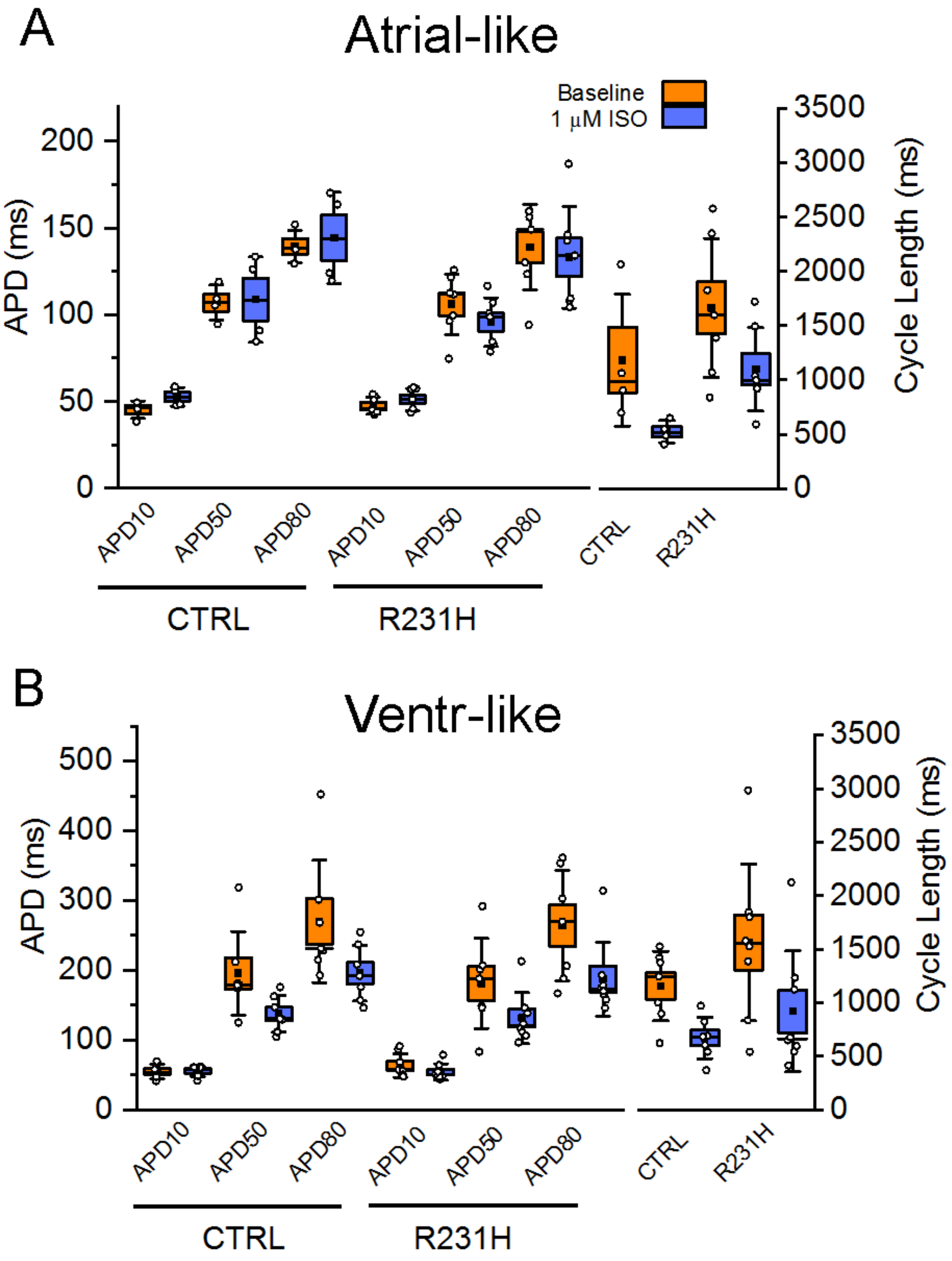

Supplementary Figure 3. Beta-adrenergic response to isoproterenol in R231H$^{+/-}$ iPSC-CMs is similar to control (CTRL) cells.

KCNQ1 subunits play an important role in APD shortening in ventricle in response to adrenergic stimulation. This experiment tests whether heterozygous mutant R231H iPSC-CMs respond differently to adrenergic stimulation, compared to control iPSC-CMs. Our data indicate that the response to isoproterenol is similar between mutant and control iPSC-CMs. APD and cycle length values for atrial-like iPSC-CMs from CTRL patient (n=4 independent cells) and R231H$^{+/-}$ patient (n=7) (**a**) and ventricular-like iPSC-CMs from CTRL patient (n=7) and R231H$^{+/-}$ patient (n=8) (**b**) at baseline (orange) and in response to perfusion with 1 µM ISO (blue). Box plots display mean (black square), median (horizontal black line), SEM (orange/blue box border), SD (error bar). Individual data points are

shown as open circles. 2-way ANOVA was performed to test for the effects of mutation status (control vs R231H), ISO treatment (baseline, ISO) and the interaction between mutation status and isoproterenol on APD values and spontaneous beating cycle length. For atrial-like cells, APD10, 50 and 80 values were not different based on mutation status or ISO exposure. For ventricular-like cells, APD10 values were not different based on mutation status or ISO exposure. Ventricular-like APD50 values were not different based on mutation status ($p=0.96$), but differed based on ISO exposure ($p<0.001$), and not different considering the combination of mutation status and ISO exposure ($p=0.54$). Ventricular-like APD80 values were not different based on mutation status ($p=0.72$), but differed based on ISO exposure ($p<0.001$), and not different considering the combination of mutation status and ISO exposure ($p=0.46$). For atrial- and ventricular-like cells, cycle lengths were different based on mutation status ($p=0.005$) and ISO exposure ($p<0.001$), but not different considering the combination of mutation status and ISO exposure ($p=0.91$). Statistical analyses performed using SYSTAT, version 13.

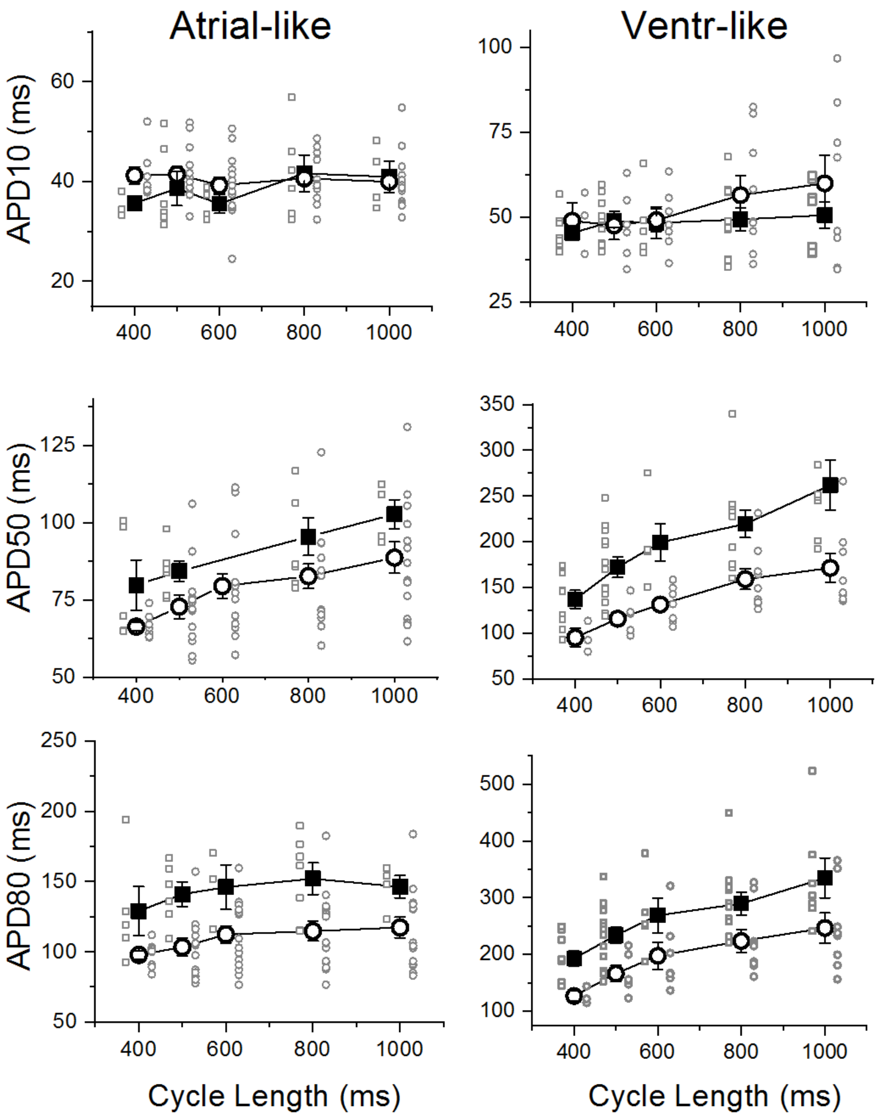

Supplementary Figure 4. Response to increasing pacing rate in R231H$^{+/-}$ iPSC-CMs is similar to control cells. An important physiological role for KCNQ1 subunits is to facilitate the shortening of action potential duration (APD) in response to increasing pacing rates (i.e., decreasing cycle length). This experiment tests whether heterozygous mutant R231H iPSC-CMs respond differently to increasing pacing rates, compared to control iPSC-CMs. Our data indicate that in spite of the gain-of-function effect (manifested by shorter baseline APD), the response to increasing pacing rate in heterozygous mutant iPSC-CMs is similar to control. Mean APD values as a function of pacing rate for atrial-like and ventricular-like iPSC-CMs derived from control patient (black squares) and R231H$^{+/-}$ patient (white circles) are displayed alongside individual data points (control, grey squares and R231H$^{+/-}$, grey circles). Data represent mean ± SEM. 2-way ANOVA was performed to test for the effects of mutation status (control vs R231H), pacing rate (1000, 800, 600, 500, 400) and the interaction between mutation

status and pacing rate on APD values. Atrial APD50, ventricular APD50 and ventricular APD80 values were significantly different between control and R231H ($p<0.001$) and across the pacing rates ($p<0.001$). However, when considering mutation status and pacing rate, the values were not significant ($p=0.90$, $p=0.66$, $p=0.99$, respectively). Atrial APD80 values were significant between control and R231H ($p<0.001$), but not different across pacing rates ($p=0.22$). Atrial APD10 and ventricular APD10 values were not different between control and R231H, nor different across pacing rates. Statistical analyses performed using SYSTAT, version 13.

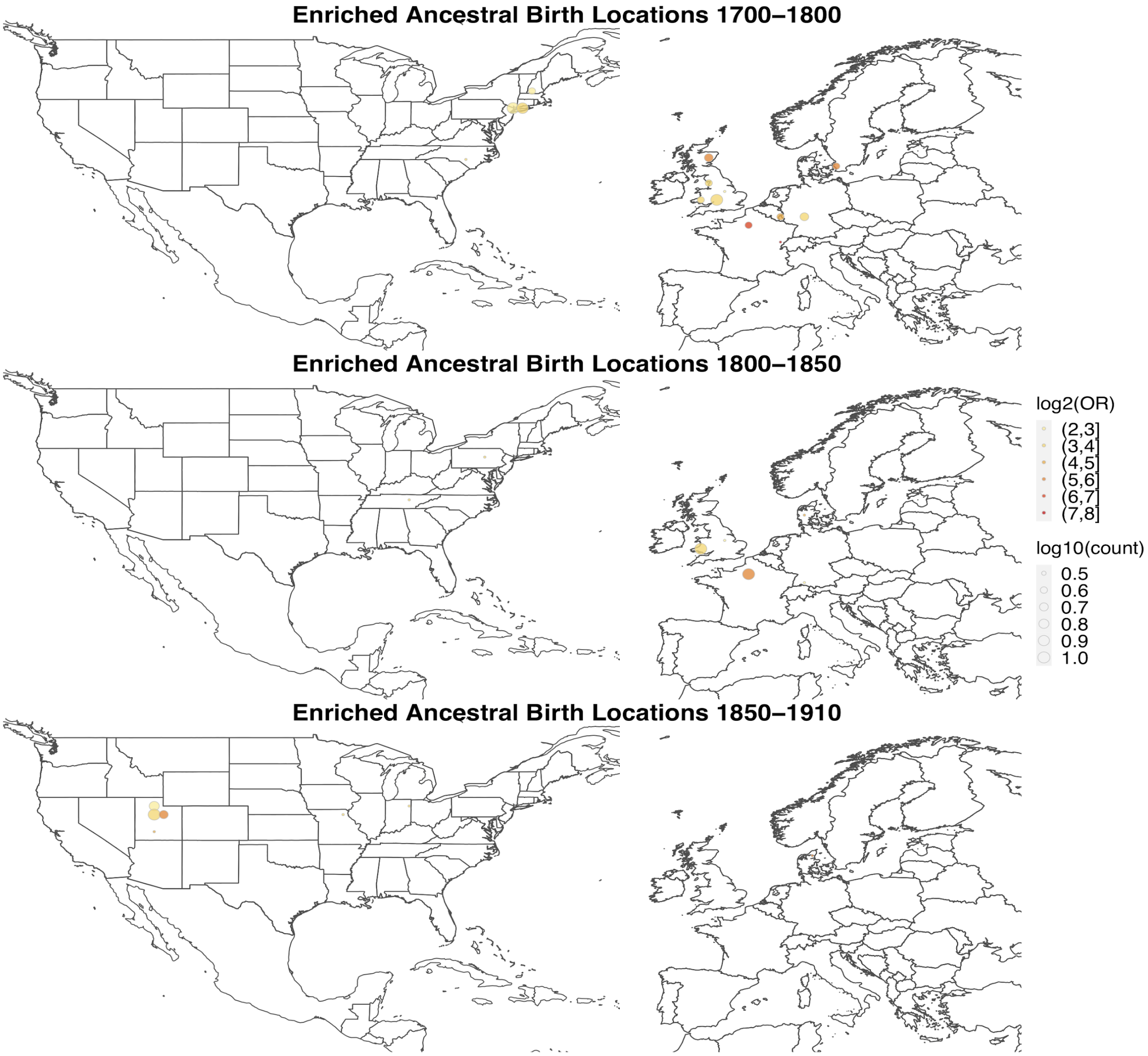

Supplementary Figure 5. Ancestral birth location enrichment maps for putative carriers of AF risk allele.

OR $\geq$ 4 for each location, with at least 2 unique family trees. Locations are rounded to the nearest latitude and longitude. Color indicates odds ratio, size indicates number of samples per location within a given time period. The ancestral birth location enrichment maps for putative carriers faithfully recapitulate the features observed for the genetic matches.

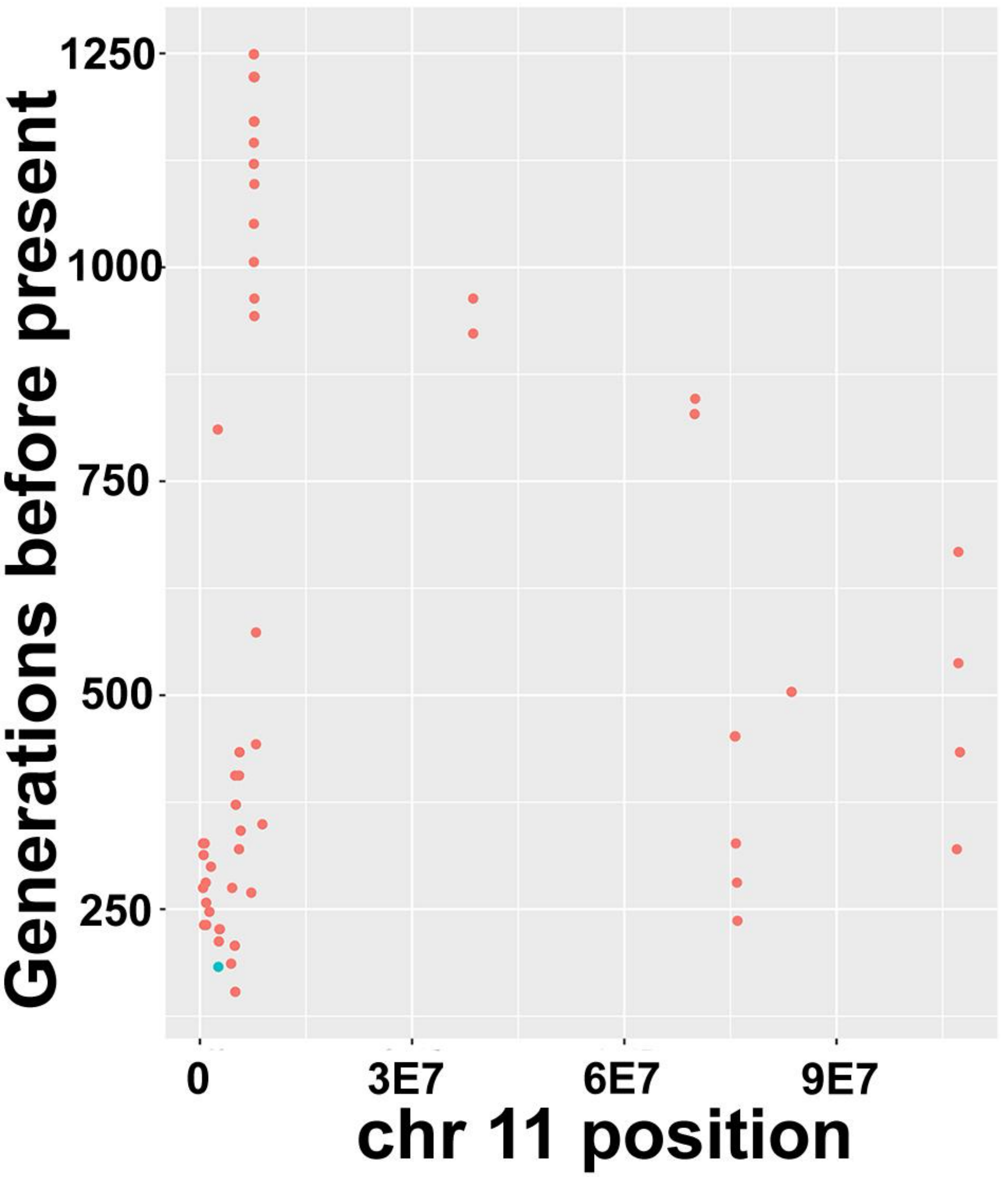

Supplementary Figure 6. ***KCNQ1*** **R231H allele is part of a cluster of recent mutations.**

The x-axis shows the position along Chromosome 11 and the y-axis shows the estimated allele age for each variant that is heterozygous in 2 whole-genome sequenced *KCNQ1* R231H risk allele carriers but absent in the CEU 1000 Genomes samples. The R231H allele is shown in light blue, and appears to be part of a cluster of alleles of recent origin.

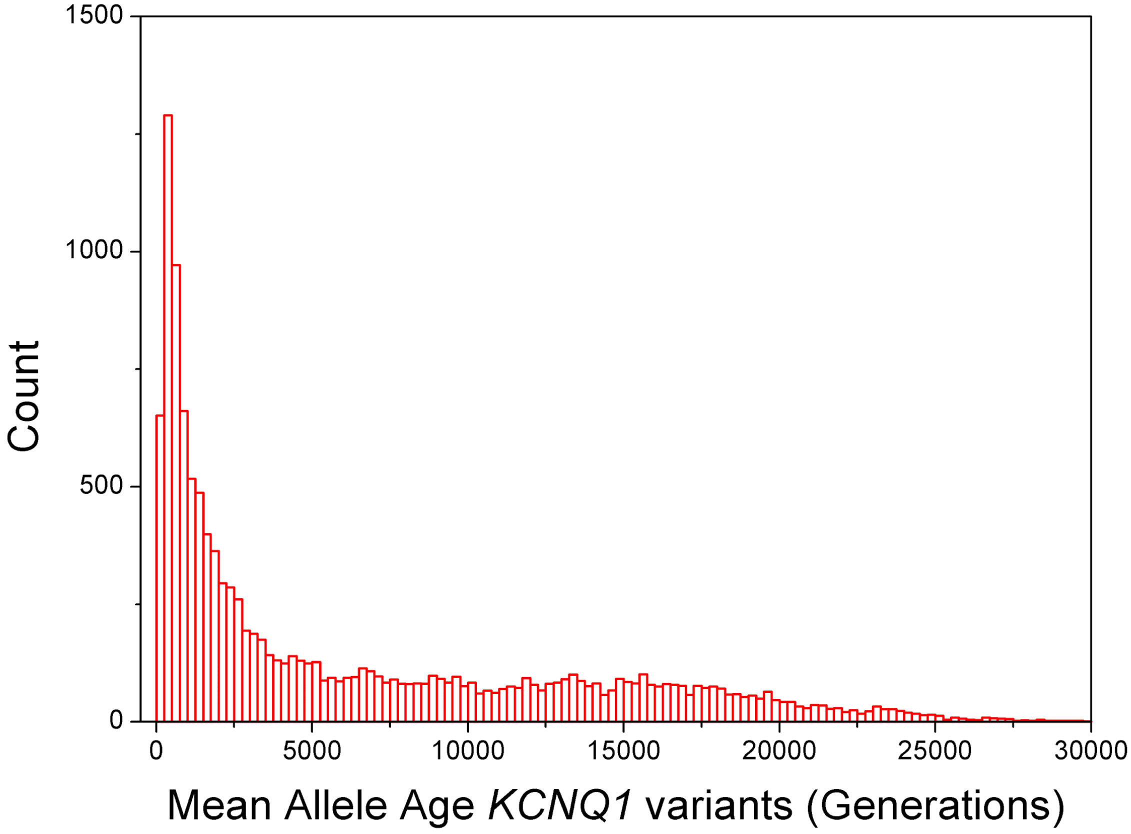

Supplementary Figure 7. Estimated age of other *KCNQ1* variants.

Histogram of GEVA-estimated age of single nucleotide variants across the *KCNQ1* locus. Graph generated using data from 1000 Genomes and Simons Genome Diversity Project as analyzed by The Atlas of Variant Age online database: https://human.genome.dating/. Each bin corresponds to 200 generations. The AF risk allele (estimated at 182 generations) would fall into the first bin and thus is among the youngest variants across *KCNQ1*.

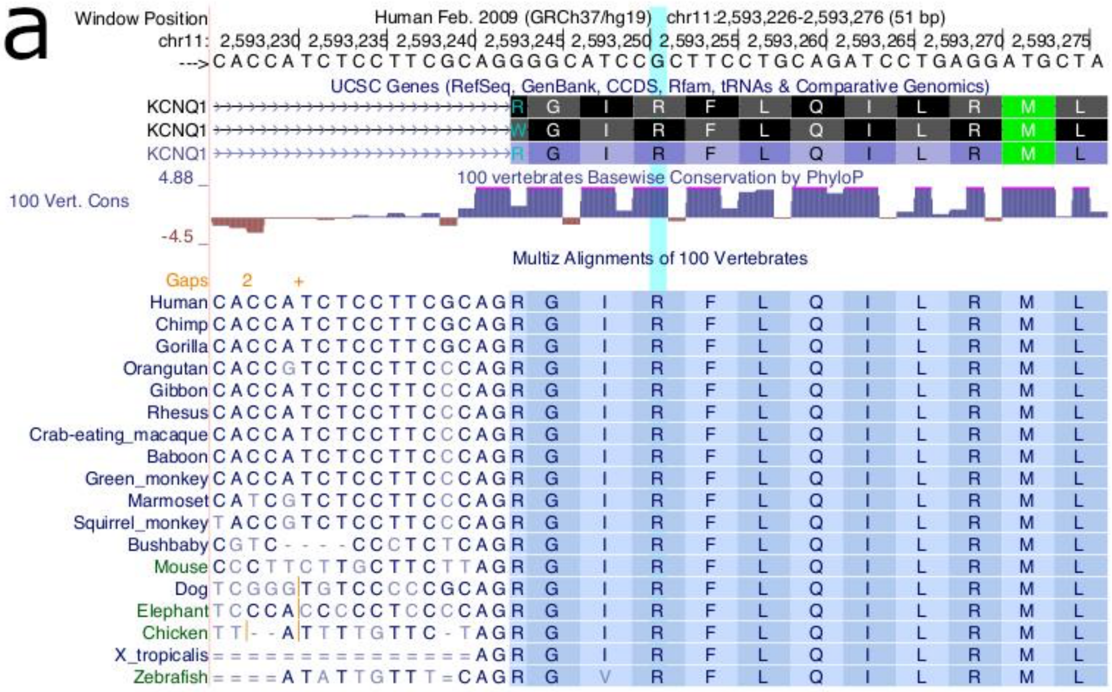

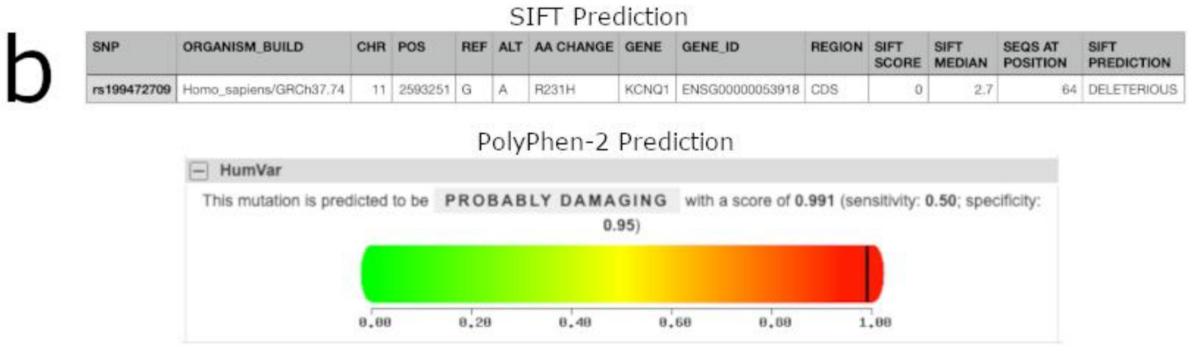

SNP	ORGANISM_BUILD	CHR	POS	REF	ALT	AA CHANGE	GENE	GENE_ID	REGION	SIFT SCORE	SIFT MEDIAN	SEQS AT POSITION	SIFT PREDICTION
rs199472709	Homo_sapiens/GRCh37.74	11	2593251	G	A	R231H	KCNQ1	ENSG00000053918	CDS	0	2.7	64	DELETERIOUS

Supplementary Figure 8. Conservation of *KCNQ1* position 231 amino acid.

a Multiple alignment of primates and other vertebrates at the KCNQ1 variant locus (highlighted light blue) shows that the arginine is highly conserved across species, further indicating the likely deleteriousness of a histidine substitution. Created using the UCSC Genome browser[12] (http://genome.ucsc.edu) with the human genome assembly Feb. 2009 (GRCh37/hg19). This multiple alignment may be viewed interactively at: https://genome.ucsc.edu/s/hateley/KCNQ1%20R231%20conservation.

b SIFT prediction (score:0, median:2.7, prediction:deleterious) and PolyPhen-2 prediction (model: "HumVar", prediction: "probably damaging", score: 0.991, sensitivity: 0.50, specificity: 0.95) provide supporting *in silico* evidence of amino acid substitution pathogenicity.

SUPPLEMENTARY TABLES

Supplementary Table 1. List of genes in linkage disequilibrium with *KCNQ1* AF risk allele.

Gene	Chromosome	Position (hg19)	Variant	Protein change	gnomAD2.1 frequency	gnomAD 3.1 frequency	pVAAST p-values	pVAAST LOD Score	rank
KCNQ1	11	2593251	G->A	R->H	0.000032	novel	7.00E-07	3.82	1
LRRC56	11	553971	G->A	G->R	0.001422	0.001131	9.00E-07	3.83	2
PNPLA2	11	824042	C->T	L->F	0.000799	0.000756	1.70E-06	3.80	3
OR52L1	11	6007218	G->A	Q->*	novel*	novel*	7.32E-06	2.11	4
CYB5R2	11	7690873	C->T	V->M	0.030050	0.061940	1.34E-05	2.17	5
PPFIBP2	11	7673029	T->C	M->T	0.037560	0.081340	1.47E-05	2.17	6
UEVLD	11	18600283	T->C	M->V	0.020000	0.037130	6.35E-05	2.67	9
EHF	11	34668175	C->T	A->V	0.028810	0.025190	2.00E-04	1.16	12
NELL1	11	20959394	C->T	R->W	0.044170	0.038090	4.31E-04	1.24	16

*Multiple stop-gain mutations within 20bp of this stop-gain

Supplementary Table 2. Definitions of selected terms used in manuscript.

Term	Definition
Population	A general term we use throughout the manuscript to refer to various sets of individuals in our analyses.<br>In our descriptions of genetic ancestry estimates, namely “genetic ethnicity estimates” and “Genetic Community assignments,” we use the term “population” to refer to the broader group of individuals in the reference set to which our samples share genetic similarity, to differentiate from the individual samples themselves or their direct relatives.<br>As all humanity is related along a continuous scale of diversity, subsetting individuals into populations is merely a useful fabrication for containing complex human history within categories for measurement purposes. Methods in this study do not intend, nor would they be able, to indicate any discrete biological distinction between populations or ancestries.
The original five	The AncestryDNA genotyped samples of 5 AF-risk allele carriers from the large family pedigree.
Genetic matches	All samples sharing at least one IBD chromosome segment > 6 centimorgans anywhere in the genome with at least one of the original five subjects.
Unphased-IBD-at-locus	Samples sharing > 1 cM unphased IBD across the region spanning the *KCNQ1* locus with at least one of the original five subjects.
Putative carriers	Samples that are likely carriers of the R231H risk variant, due to sharing unphased-IBD-at-locus with all of the original five subjects along the chromosome segment spanning *KCNQ1*
Genetic ethnicity estimate	Term used by AncestryDNA to designate the estimate of genetic similarity to a reference panel comprising representatives of continental and subcontinental populations of varying degrees of resolution.<br>Estimates rely upon similarity to reference samples, and are not indicative of ground truth regions of origin.
Genetic Community assignment	Term used by AncestryDNA to designate the estimate of recent population ancestry via genetic similarity to sets of samples sharing recent ancestry and annotated using linked aggregated historical and genealogical records.

Supplementary Table 3. IBD amongst original five subjects, with corresponding inferred meiosis events and relationships.

Sample 1	Sample 2	Total cM IBD	Inferred separating meioses	cM IBD across locus
C	D	2493.04	2	5.32
C	E	1689.65	3	44.35
D	E	1524.09	3	5.32
A	E	108.08	7	14.43
A	B	40.14	9	10.40
A	C	32.35	9	14.43
B	C	25.58	9	10.29
B	E	23.36	9	10.40
A	D	14.24	10	4.67
B	D	<6	11	1.23

Inferred relationships
2 = full sibling
3 = half sibling; avuncular; grandparent; double 1st cousin
7 = 2nd cousin, 1x removed; half second cousin; 1st cousin, 3x removed; half 1st cousin, 2x removed
9 = 3rd cousin, 1x removed; distant cousin
10, 11 = distant cousin

Supplementary Table 4. Broad-scale genetic ancestry estimates of the original five subjects.

Group: Original five subjects					
	Mann-Whitney U statistics				
Region	U statistic	P-value	In-group median estimate	Out-group median estimate	Mean (and range) estimate %
Norway	4.3e7	8.1e-3	12%	0%	12 (0-26)
England, Wales & Northwestern Europe	4.2e7	1.9e-2	64%	45%	66 (52-84)
Sweden	3.9e7	3.8e-2	2%	0%	6 (0-13)
Ireland & Scotland		not significant			12 (3-20)
Germanic Europe		not significant			4 (0-7)

Supplementary Table 5. Significant broad-scale genetic ancestry estimates of the Mountain West Mormon Pioneers community, genetic matches, and putative carriers.

Group: Mountain West Mormon Pioneers				
Region	U statistic	P-value	In-group median estimate	Out-group median estimate
Norway	1.8e12	(p < 0.001)	4%	0%
England, Wales & Northwestern Europe	1.8e12		62%	44%
Sweden	1.8e12		4%	0%
Germanic Europe	1.4e12		4%	2%
**Group: Genetic matches (IBD anywhere to any of the original five subjects)**				
Region	U statistic	P-value	In-group median estimate	Out-group median estimate
Norway	8.9e11	(p < 0.001)	2%	0%
England, Wales & Northwestern Europe	1.0e12		60%	44%
Sweden	8.6e11		1%	0%
Ireland & Scotland	8.6e11		15%	12%
Germanic Europe	8.0e11		3%	2%
**Group: Putative carriers**				
Region	U statistic	P-value	In-group median estimate	Out-group median estimate
England, Wales & Northwestern Europe	2.7e8	<0.001	68%	45%
Norway	2.1e8	0.0071	3%	0%
Sweden	2.0e8	0.0182	2%	0%

Supplementary Table 6. AncestryDNA Genetic Communities over-represented in study groups.

Group: Original five subjects				
Genetic Community	Group samples in community	P-value	P-adjusted	Fold over-representation
Mountain West Mormon Pioneers	5	1.05E-08	1.13E-05	39.41
Group: Putative carriers				
Genetic Community	Group samples in community	P-value	P-adjusted	Fold over-representation
Mountain West Mormon Pioneers	21	1.08E-26	1.16E-23	26.7
Group: Genetic matches (IBD anywhere to any of the original five subjects)				
Genetic Community	Group samples in community	P-value	P-adjusted	Fold over-representation
Hjorring	362	2.79E-150	2.99E-147	5.66
Cardston, Alberta Mormon Pioneers	353	1.17E-143	1.26E-140	5.54
Mountain West Mormon Pioneers	19525	0	0	5.47
West Hjorring	349	1.90E-109	2.04E-106	4.28
Aalborg	347	2.04E-105	2.19E-102	4.16
North Jutland	1619	0	0	4.11
S. Åsnes, Western Grue, Kongsvinger	563	6.21E-87	6.66E-84	2.59
Denmark	3330	0	0	2.54
Southeastern Hedmark	755	6.72E-95	7.21E-92	2.33
Southwestern Hedmark	215	6.36E-28	6.83E-25	2.31
East Jutland	422	6.48E-51	6.95E-48	2.27
Viborg & Hjorring	352	1.80E-40	1.93E-37	2.21
Lancashire & Greater Manchester	1308	3.75E-129	4.03E-126	2.1
Southeast Oppland & Hedmarken	603	1.65E-43	1.77E-40	1.84
West Midlands County	494	1.38E-35	1.48E-32	1.84
S. Hedmark, Eastern Akershus, S. Oppland	1872	1.13E-129	1.21E-126	1.84

Lolland, Falster & Møn	93	7.16E-08	7.68E-05	1.82
RI & Southeastern MA Settlers	5011	0	0	1.8
Wigan, Bolton & Warrington	230	1.69E-16	1.81E-13	1.8
Østfold, Oslo & Southern Akershus	317	6.91E-21	7.41E-18	1.76
West Midlands	1801	8.33E-110	8.94E-107	1.76
Nordre & Søndre Land, N. Toten, Ringsakker	236	1.16E-15	1.24E-12	1.75
E. Kentucky & SW Virginia Settlers	1012	1.02E-59	1.09E-56	1.74
Lancashire	662	1.32E-39	1.42E-36	1.74
MA, VT, RI & CT Settlers	1896	1.22E-106	1.30E-103	1.72
S. Ringsaker & Hamar & Northern Stange	147	3.70E-09	3.97E-06	1.67
New England & Eastern Great Lakes Settlers	11426	0	0	1.66
Tennessee Upper Cumberland Settlers	628	6.79E-32	7.28E-29	1.65
Central Hedmark	134	4.47E-08	4.80E-05	1.65
Kentucky Eastern Pennyroyal Settlers	504	1.20E-25	1.29E-22	1.64
Cheshire, Merseyside & South Lancashire	897	3.81E-44	4.08E-41	1.64
Toten	182	2.88E-10	3.10E-07	1.64
Arkansas River Valley Settlers	117	5.03E-07	5.40E-04	1.63
The Midlands, England	4768	1.63E-219	1.75E-216	1.62
Northeastern States Settlers	16010	0	0	1.61
MA, VT & NH Settlers	4004	2.51E-172	2.69E-169	1.6
Jutland, Zealand, Funen, Lolland & Falster	1055	1.65E-44	1.77E-41	1.58
Southeastern Kentucky Settlers	776	2.69E-31	2.89E-28	1.56
New York City & Long Island Settlers	3190	1.15E-122	1.23E-119	1.55
Northwest Arkansas Settlers	190	2.34E-08	2.51E-05	1.53

Supplementary Table 4-6 Description. Enriched broad- and fine-scale population genetic ancestries across study groups.

AncestryDNA's genetic ethnicity estimate mean values for the original five subjects (Suppl Table 4) closely resemble the significantly associated genetic ethnicities for the samples making up the MWMP Genetic Community, the genetic matches, and the putative carriers (Suppl Table 5). Over-represented AncestryDNA Genetic Communities for each group are displayed in Suppl. Table 6. All original five subjects received the Genetic Community assignment "Mountain West Mormon Pioneers" (MWMP); no other Genetic Community was assigned jointly to all the original five subjects. MWMP was the only significant Genetic Community in the putative carrier group, with 21/31 samples receiving the assignment. The genetic matches group showed ~5.5-fold overrepresentation for the MWMP community compared to the overall database. While many communities were overrepresented $\geq$ 1.5-fold in the genetic matches group, the MWMP community showed one of the highest enrichments in combination with the most in-group samples assigned to a community. Interestingly, the majority of the other overrepresented Genetic Communities are of Danish origin.

Supplementary Table 7. Sanger results of selected samples.

Relationship to the original five subjects	Number of samples sequenced	Genotype at risk locus
Unphased-IBD-at-locus to all of the original five	3	A/G
Unphased-IBD-at-locus to one of the original five	10	G/G
Genetic match without IBD at locus	23	G/G
Unrelated to any of the original five	8	G/G

Of the samples available for Sanger sequencing, all those purported to be carriers were validated as heterozygous at the risk locus. All other samples were confirmed to be homozygous major at the risk locus.

Supplementary Table 8. Comparable substitute software suggestions.

Procedure	Suggested Software	Parameters / Notes
Phasing	Beagle[13]<br>or<br>Eagle[14]	We suggest two software options for phasing. Our internal phasing software is based off of Beagle and as such is most similar to Beagle. Eagle is another great option that is quite fast and recommended for use with the suggested GERMLINE IBD detection software.<br><br>For the phasing reference panel, we recommend either 1000 Genomes Project phase 3 fully phased haplotypes[5] or Haplotype Reference Consortium[3] panel.<br><br>Pseudocode explaining Underdog updates to Beagle can be found in Appendix A and B (pg. 36-43) of the Ancestry DNA Matching White Paper[10].<br><br>See Beagle website for latest software and manual: http://faculty.washington.edu/browning/beagle/beagle.html<br><br>See Eagle website for latest software and manual: https://alkesgroup.broadinstitute.org/Eagle/
IBD Detection	GERMLINE[15]	For detecting genetic matches:<br>- 96 SNP (~1 cM) match-seed window (-bits 96)<br>- Extend to homozygous mismatch<br>- Use unphased genotypes when extending from match-seed<br>- Extend past seed window (-w-extend)<br>- Report IBD segments > 6cM (-min_m 6)<br>- err_hom 0, -err_het 0<br>- For filtering out likely IBS segments, see pseudocode in Appendix C (pg. 44) of the Ancestry DNA Matching White Paper[10]. Filtering requires a large set of reference individuals.<br><br>Modifications to detect small segments across risk

		locus (see Supplementary Methods):<br>- 50 SNP (~0.5 cM) match-seed window (-bits 50)<br>- Report IBD segments > 1 cM or > 0.5cM<br>- Requires supporting evidence via sharing to all confirmed cases in IBD network<br><br>See GERMLINE website for latest software and manual: http://gusevlab.org/projects/germline/
Genetic Ancestry Inference	RFMix[16]	For a diverse reference panel we suggest using 1000 Genomes[5] and Human Genetic Diversity Project[17] panels.<br><br>RFMix can be run with the parameters used in benchmarking in the AncestryDNA ARCHes paper[18].<br><br>See RFMix website for latest software and manual: https://github.com/slowkoni/rfmix

Supplementary Table 9. Primers used for *KCNQ1* mutation.

Species	Gene	Target	Forward Primer Name	Forward Primer Sequence (5' ->3')	Reverse Primer Name	Reverse Primer Sequence (5' ->3')	Purpose	Amplicon Size (bp)
Human	KCNQ1	Exon 5	KCNQ1-Ex5F	GGGACACCCATGCCATC	KCNQ1-Ex5R	CTAGTGTGGGCTGCTCTGC	PCR amplifcation for Sanger	260

SUPPLEMENTARY REFERENCES

1. Karczewski, K. J. et al. The mutational constraint spectrum quantified from variation in 141,456 humans. *Nature* **581**, 434–443 (2020).
2. Karczewski, K. J. et al. The ExAC browser: displaying reference data information from over 60 000 exomes. *Nucleic Acids Res.* **45**, D840–D845 (2017).
3. McCarthy, S. et al. A reference panel of 64,976 haplotypes for genotype imputation. *Nat. Genet.* **48**, 1279–1283 (2016).
4. NHLBI GO Exome Sequencing Project (ESP), Seattle, WA. Exome Variant Server. https://evs.gs.washington.edu/EVS/.
5. Auton, A. et al. A global reference for human genetic variation. *Nature* **526**, 68–74 (2015).
6. Glusman, G., Caballero, J., Mauldin, D. E., Hood, L. & Roach, J. C. Kaviar: an accessible system for testing SNV novelty. *Bioinformatics* **27**, 3216–3217 (2011).
7. Komaki, S. et al. iMETHYL: an integrative database of human DNA methylation, gene expression, and genomic variation. *Hum. Genome Var.* **5**, 18008 (2018).
8. Xue, J., Lencz, T., Darvasi, A., Pe'er, I. & Carmi, S. The time and place of European admixture in Ashkenazi Jewish history. *PLOS Genet.* **13**, e1006644 (2017).
9. Curtis, R. et al. Genetic Communities White Paper: Predicting fine-scale ancestral origins from the genetic sharing patterns among millions of individuals. https://www.ancestry.com/cs/dna-help/communities/whitepaper.
10. Wang, Y. et al. AncestryDNA Matching White Paper: Discovering genetic matches across a massive, expanding genetic database. https://www.ancestry.com/corporate/sites/default/files/AncestryDNA-Matching-White-Paper.pdf (2016).
11. Guo, Y. et al. Illumina human exome genotyping array clustering and quality control. *Nat. Protoc.* **9**, 2643–2662 (2014).
12. Kent, W. J. et al. The Human Genome Browser at UCSC. *Genome Res.* **12**, 996–1006 (2002).
13. Browning, S. R. & Browning, B. L. Rapid and Accurate Haplotype Phasing and Missing-Data Inference for Whole-Genome Association Studies By Use of Localized Haplotype Clustering. *Am. J. Hum. Genet.* **81**, 1084–1097 (2007).
14. Loh, P.-R. et al. Reference-based phasing using the Haplotype Reference Consortium panel. *Nat. Genet.* **48**, 1443–1448 (2016).
15. Gusev, A. et al. Whole population, genome-wide mapping of hidden relatedness. *Genome Res.* **19**, 318–326 (2009).
16. Maples, B. K., Gravel, S., Kenny, E. E. & Bustamante, C. D. RFMix: a discriminative modeling approach for rapid and robust local-ancestry inference. *Am. J. Hum. Genet.* **93**, 278–288 (2013).
17. Li, J. Z. et al. Worldwide Human Relationships Inferred from Genome-Wide Patterns of Variation. *Science* **319**, 1100 (2008).
18. Noto, K. et al. Ancestry Inference Using Reference Labeled Clusters of Haplotypes. *bioRxiv* 2020.09.23.310698 (2020) doi:10.1101/2020.09.23.310698.
