# Supplementary material for: The history and geographic distribution of a KCNQ1 atrial fibrillation risk allele
Source: Nat Commun. 2021 Nov 8;12:6442. doi: 10.1038/s41467-021-26741-7 (PMC8575962; doi:10.1038/s41467-021-26741-7)
Supplement: Supplementary file 2 — Reporting Summary [file 41467_2021_26741_MOESM2_ESM.pdf]

nature research

Corresponding author(s): Martin Tristani-Firouzi

Last updated by author(s): October 16, 2021

Reporting Summary

Nature Research wishes to improve the reproducibility of the work that we publish. This form provides structure for consistency and transparency in reporting. For further information on Nature Research policies, see our Editorial Policies and the Editorial Policy Checklist.

Statistics

For all statistical analyses, confirm that the following items are present in the figure legend, table legend, main text, or Methods section.

n/a	Confirmed	
☐	☒	The exact sample size (*n*) for each experimental group/condition, given as a discrete number and unit of measurement
☐	☒	A statement on whether measurements were taken from distinct samples or whether the same sample was measured repeatedly
☐	☒	The statistical test(s) used AND whether they are one- or two-sided<br>*Only common tests should be described solely by name; describe more complex techniques in the Methods section.*
☒	☐	A description of all covariates tested
☐	☒	A description of any assumptions or corrections, such as tests of normality and adjustment for multiple comparisons
☐	☒	A full description of the statistical parameters including central tendency (e.g. means) or other basic estimates (e.g. regression coefficient) AND variation (e.g. standard deviation) or associated estimates of uncertainty (e.g. confidence intervals)
☐	☒	For null hypothesis testing, the test statistic (e.g. *F*, *t*, *r*) with confidence intervals, effect sizes, degrees of freedom and *P* value noted<br>*Give P values as exact values whenever suitable.*
☐	☒	For Bayesian analysis, information on the choice of priors and Markov chain Monte Carlo settings
☒	☐	For hierarchical and complex designs, identification of the appropriate level for tests and full reporting of outcomes
☒	☐	Estimates of effect sizes (e.g. Cohen's *d*, Pearson's *r*), indicating how they were calculated

Our web collection on statistics for biologists contains articles on many of the points above.

Software and code

Policy information about availability of computer code

Data collection	Data collection and annotation software such as the Genome Analysis ToolKit (GATK) and the Variant Effect Predictor and their use are described in the Methods. AncestryDNA sample collection and processing are described in the Supplementary Methods.
Data analysis	All data analysis software and their use are described in the Methods and Supplementary Methods and Tables. Software used or referenced: VAAST2.0, pVAAST v1, BEAGLE v3.3.2, GERMLINE v1.5.1, AncestryDNA Underdog v1, AncestryDNA Ethnicity Estimates 2019, Ancestry Community Assignment 2019, GEVA v1beta, Gephi v0.9.2, Eagle v2.4, RFMix v2, DESeq2, UCSC Genome browser, OriginPro2020b, BWA MEM, GATK, SIFT, PolyPhen-2, REVEL.

For manuscripts utilizing custom algorithms or software that are central to the research but not yet described in published literature, software must be made available to editors and reviewers. We strongly encourage code deposition in a community repository (e.g. GitHub). See the Nature Research guidelines for submitting code & software for further information.

Data

Policy information about availability of data

All manuscripts must include a data availability statement. This statement should provide the following information, where applicable:

- Accession codes, unique identifiers, or web links for publicly available datasets
- A list of figures that have associated raw data
- A description of any restrictions on data availability

To protect patient privacy, sequencing data (exome, genome, RNA-Seq) for this study has been deposited into the database of Genotypes and Phenotypes (dbGaP) under accession phs002464.v1.p1 (https://www.ncbi.nlm.nih.gov/projects/gap/cgi-bin/study.cgi?study_id=phs002464.v1.p1) and is available to users upon approval of a Data Access Request (https://dbgap.ncbi.nlm.nih.gov/aa/wga.cgi?page=login). RNA-Seq data for human iPSC lines whose expression patterns were previously corroborated against human embryonic stem cell lines was obtained here: https://www.ncbi.nlm.nih.gov/gds/?term=GSE73211. The human genome reference

sequence (GRCh37/hg19) may be downloaded from http://genome.ucsc.edu. The 1000 Genomes Project Phase 3 high coverage sequencing data of 99 CEU individuals used in the variant age analysis can be downloaded from The International Genome Sample Resource website at https://www.internationalgenome.org/data-portal/data-collection/30x-grch38. Ancestry Public Family Trees are available at the Ancestry.com Public Member Trees online database, searchable at: https://www.ancestry.com/search/collections/1030/. While AncestryDNA cannot make the genealogical and genotype data available to the academic community in light of our commitment to customer privacy, we will do our best to accommodate requests regarding methods and summary statistics for the purpose of reproducibility, subject to applicable data use policies. AncestryDNA is interested in pursuing research collaboration opportunities. Please contact Dr. Barry Starr (bstarr@ancestry.com) for methods and summary statistics requests, and for guidelines on submitting a research proposal for consideration.

Field-specific reporting

Please select the one below that is the best fit for your research. If you are not sure, read the appropriate sections before making your selection.

[x] Life sciences [ ] Behavioural & social sciences [ ] Ecological, evolutionary & environmental sciences

For a reference copy of the document with all sections, see nature.com/documents/nr-reporting-summary-flat.pdf

Life sciences study design

All studies must disclose on these points even when the disclosure is negative.

Sample size	This is an observational cohort study of a single family. For the AncestryDNA study, sample size (n=140,722) was dictated by number of available research-consented samples sharing genetic relatedness with the single family cohort samples, providing an abundance of samples for statisticalyl significant analysis.
Data exclusions	No data were excluded from this study.
Replication	*Describe the measures taken to verify the reproducibility of the experimental findings. If all attempts at replication were successful, confirm this OR if there are any findings that were not replicated or cannot be reproduced, note this and describe why.*
Randomization	*Describe how samples/organisms/participants were allocated into experimental groups. If allocation was not random, describe how covariates were controlled OR if this is not relevant to your study, explain why.*
Blinding	*Describe whether the investigators were blinded to group allocation during data collection and/or analysis. If blinding was not possible, describe why OR explain why blinding was not relevant to your study.*

Reporting for specific materials, systems and methods

We require information from authors about some types of materials, experimental systems and methods used in many studies. Here, indicate whether each material, system or method listed is relevant to your study. If you are not sure if a list item applies to your research, read the appropriate section before selecting a response.

Materials & experimental systems

n/a	Involved in the study	
[ ]	[x]	Antibodies
[ ]	[x]	Eukaryotic cell lines
[x]	[ ]	Palaeontology and archaeology
[x]	[ ]	Animals and other organisms
[ ]	[x]	Human research participants
[ ]	[x]	Clinical data
[x]	[ ]	Dual use research of concern

Methods

n/a	Involved in the study	
[x]	[ ]	ChIP-seq
[x]	[ ]	Flow cytometry
[x]	[ ]	MRI-based neuroimaging

Antibodies

Antibodies used

Set 1:
Mouse anti-Tra-1-60 IgM (Thermofisher Scientific, MA1-023)
Goat anti-mouse IgM-AF647 (Thermofisher Scientific, A21238)
Mouse IgM isotype control (Thermofisher Scientific, 14-4752-82)

Rabbit anti-Nanog IgG (Thermofisher Scientific, PA1-097)
Goat anti-rabbit IgG-AF488 (Thermofisher Scientific, A11034)
Rabbit IgG isotype control (Thermofisher Scientific, 02-6102)

Set 2:
Mouse anti-OCT4 IgG1 (Thermofisher Scientific, MA1-104)
Goat anti-mouse IgG-AF488 (Thermofisher Scientific, A11001)

	Mouse IgG1 isotype control (Thermofisher Scientific, MA1-10405)<br><br>Rat anti-SOX2 IgG2a kappa (Thermofisher Scientific, 14-9811-82)<br>Goat anti-rat IgG-AF555 (Thermofisher Scientific, A21434)<br>Rat IgG2a kappa isotype control (Thermofisher Scientific, 14-4321-82)
Validation	*Describe the validation of each primary antibody for the species and application, noting any validation statements on the manufacturer's website, relevant citations, antibody profiles in online databases, or data provided in the manuscript.*

Eukaryotic cell lines

Policy information about cell lines

Cell line source(s)	Induced pluripotent stem cell lines derived from patient peripheral blood mononuclear cells
Authentication	Cell lines were authenticated using short tandem repeat (STR) analysis across 24 chromosomal regions
Mycoplasma contamination	All lines were tested for mycoplasma contamination and confirmed negative.
Commonly misidentified lines (See ICLAC register)	*Name any commonly misidentified cell lines used in the study and provide a rationale for their use.*

Human research participants

Policy information about studies involving human research participants

Population characteristics	This is an observational, retrospective analysis of a large family with autosomal dominant (and variable penetrance) arrhythmia, primarily of European origin, male and female, ages 13-80. AncestryDNA participants were > 18 years of age and a subset of the AncestryDNA customer population.
Recruitment	Participants were recruited by chart review of records linked to the Utah Population Database, as described in the Methods section. AncestryDNA samples were selected from customers consented to participate in AncestryDNA's Human Diversity Project and population-level results reflect the genetic diversity present in the AncestryDNA customer base.
Ethics oversight	Institutional Review Board of University of Utah, Intermountain Medical Center, and AncestryDNA.

Note that full information on the approval of the study protocol must also be provided in the manuscript.

Clinical data

Policy information about clinical studies

All manuscripts should comply with the ICMJEguidelines for publication of clinical research and a completedCONSORT checklist must be included with all submissions.

Clinical trial registration	*Provide the trial registration number from ClinicalTrials.gov or an equivalent agency.*
Study protocol	*Note where the full trial protocol can be accessed OR if not available, explain why.*
Data collection	*Describe the settings and locales of data collection, noting the time periods of recruitment and data collection.*
Outcomes	*Describe how you pre-defined primary and secondary outcome measures and how you assessed these measures.*
